# Diversity and host interaction of the gut microbiota in specific pathogen-free pigs

**DOI:** 10.3389/fmicb.2024.1402807

**Published:** 2024-05-10

**Authors:** Mingxing Wen, Shuangshuang Chen, Yali Zhang, Yan Liu, Chuang Tang, Jinwei Zhang, Jing Sun, Xiaokai Li, Yuchun Ding, Lu Lu, Keren Long, Yong Nie, Xuewei Li, Mingzhou Li, Liangpeng Ge, Jideng Ma

**Affiliations:** ^1^State Key Laboratory of Swine and Poultry Breeding Industry, College of Animal Science and Technology, Sichuan Agricultural University, Chengdu, China; ^2^Chongqing Academy of Animal Sciences, Chongqing, China; ^3^National Center of Technology Innovation for Pigs, Chongqing, China; ^4^Ministry of Agriculture Key Laboratory of Pig Sciences, Chongqing Key Laboratory of Pig Sciences, Chongqing, China; ^5^College of Engineering, Peking University, Beijing, China

**Keywords:** specific pathogen-free pigs, metagenomics, transcriptomics, gut microbiota, host-microbial interactions

## Abstract

Pigs are widely used as animal models in various studies related to humans. The interaction between the gut microbiota and the host has significant effects on the host’s health and disease status. However, although there have been many studies investigating the pig gut microbiota, the findings have been inconsistent due to variations in rearing conditions. Interactions between the gut microbiota and host have not been fully explored in pigs. Specific pathogen-free (SPF) pigs are ideal non-primate large animals to study the interactions between the gut microbiota and the host. In this study, we performed high-throughput sequencing analysis of the gut microbiota and the gut tissue transcriptome of six SPF pigs to provide a systematic understanding of the composition, function, and spatial distribution of gut microbiota in SPF pigs. We identified significant differences in microbial diversity and functionality among different gastrointestinal tract sites. Metagenomics data analysis revealed significant differences in alpha diversity and beta diversity of microbiota in different gastrointestinal sites of SPF pigs. Additionally, transcriptomic data indicated significant differences in gene expression as well as KEGG and GO functional enrichment between the small intestine and large intestine. Furthermore, by combining microbial metagenomics and host transcriptomics analyses, specific correlations were found between gut microbiota and host genes. These included a negative correlation between the *TCN1* gene and *Prevotella dentalis*, possibly related to bacterial metabolic pathways involving vitamin B12, and a positive correlation between the *BDH1* gene and *Roseburia hominis*, possibly because both are involved in fatty acid metabolism. These findings lay the groundwork for further exploration of the co-evolution between the microbiota and the host, specifically in relation to nutrition, metabolism, and immunity. In conclusion, we have elucidated the diversity of the gut microbiota in SPF pigs and conducted a detailed investigation into the interactions between the gut microbiota and host gene expression. These results contribute to our understanding of the intricate dynamics between the gut microbiota and the host, offering important references for advancements in life science research, bioproduct production, and sustainable development in animal husbandry.

## Introduction

Animal models play a significant role in studying human development and disease, and in the search for effective therapies and vaccines. Compared with rodent models, pigs are closer to humans in anatomical size and structure, and in immunological, genomic, and physiological processes (Lunney, 2007; Meurens et al., 2012; Swindle et al., 2012; Gerdts et al., 2015; Gutierrez et al., 2015; Klymiuk et al., 2016; Pabst, 2020), making them advantageous for translational and clinical research applications. Additionally, pigs have several advantages over primate models and other animal models, including shorter generational intervals, larger litter sizes, and ease of genome editing. Therefore, pigs hold tremendous potential for the generation of biomedical models to study human developmental processes (Swindle et al., 1996; Ferenc et al., 2014; Jiang et al., 2019), congenital diseases (Hu et al., 2016; Perleberg et al., 2018), and pathogen response mechanisms (Amaral et al., 2020; Ramos et al., 2020).

Recent studies using model pigs have generally used animals raised under conventional (farm) conditions (Qiao et al., 2021; Song et al., 2022; Narduzzi et al., 2023; Rupp et al., 2023). However, research on the gut microbiota of farm-raised pigs can be influenced by various factors, such as pathogen contamination, dietary differences, environmental factors, and the use of drugs and antibiotics. Specific pathogen-free (SPF) pigs are raised in an environment free from pathogenic microorganisms, resulting in a relatively stable and clean gut microbial community. Compared with germ-free and gnotobiotic pigs, SPF pigs have lower housing facility requirements but these are still higher than those used for conventional rearing (Safron and Gonder, 1997). The gut microbiota plays a critical role in host immune training (Donald and Finlay, 2023; Koren et al., 2023), food digestion (Caminero et al., 2019; García-Pérez et al., 2024), regulation of intestinal endocrine function and neural signal transmission (Mazzoli and Pessione, 2016; Zhao et al., 2020), drug action and metabolism (Wilson and Nicholson, 2017; Kolli and Roy, 2023), toxin clearance (Wang et al., 2019), and production of various compounds that can influence the host (Kudjordjie et al., 2019; Tennoune et al., 2022). Pigs share many similarities with humans in terms of gut microbiota composition and function (Roura et al., 2016), making SPF pigs an ideal non-primate large animal model for studying the interaction between gut microbiota and the host (Zhou et al., 2021). Although research into the gut microbiota of pigs has progressed rapidly in recent years, resulting in the construction and improvement of pig gut microbiota catalogs (Xiao et al., 2016; Chen et al., 2021a), there are no unified standard procedures and these studies have used different rearing conditions and pig breeds, which has led to inconsistent results (Yang et al., 2016; Holman et al., 2017; Wang et al., 2019; Chen et al., 2021b). Additionally, the interaction between the gut microbiota and host gene expression has not been fully considered. Therefore, there is an urgent need to establish a standardized gut microbiota profile of SPF pigs as an important reference for the livestock industry, life science research, and bioproduct production.

Here, we collected the gastrointestinal tract (GIT) contents and intestinal tissues of SPF Bama females in which the gut microbiota had reached a stable and plateau phase (Frese et al., 2015; Dong et al., 2023), and performed high-throughput sequencing analysis. We comprehensively revealed the diversity and functional differences of the gut microbiota in different GIT sites of SPF pigs. Furthermore, we investigated potential functional interactions between the gut microbiota and the host. This provides a foundation for further research into the co-evolutionary mechanisms between microbiota and the host. In summary, through in-depth investigation of changes in gut microbiota composition, function, and host gene expression, a better understanding of gut microbiota ecology in SPF pigs can be determined and the interaction between the gut microbiota and the host can be explored to provide a scientific basis for improving pig health and productivity.

## Materials and methods

### Ethics approval

This study was approved by the Experimental Animal Ethics Committee, Chongqing Academy of Animal Science, under reference number XKY-No. 20210606.

### Sample collection

To investigate differences in the gut microbiota of SPF pigs and their interaction with the host, we selected Bama females provided by Chongqing Academy of Animal Science (Chongqing, China) that were disease-free, unrelated, in good physical condition, and normal in appearance. These females were pregnant and tested negative for the placental transmission diseases, African swine fever, porcine pseudorabies, porcine parvovirus disease, and porcine reproductive and respiratory syndrome, and for other serum-specific pathogens, such as African swine fever, foot-and-mouth disease, Japanese encephalitis, porcine circovirus, swine influenza, and porcine brucellosis.

**Figure 1:**
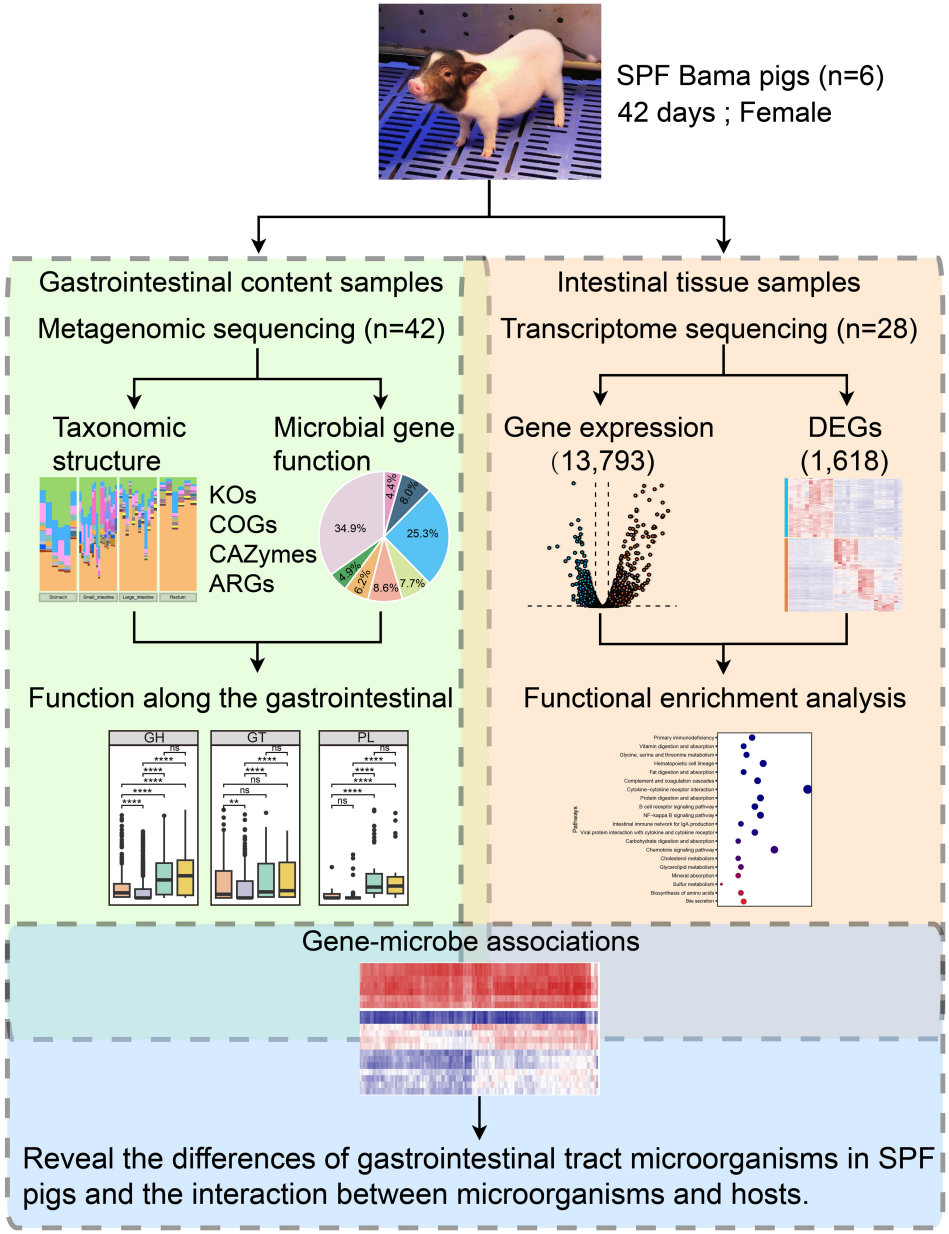
Overview of the integrated multi-omics workflow combining metagenomics and transcriptomics. We selected six 42-day-old SPF Bama females as the study subjects and extracted gastrointestinal content samples (*n* = 42) and intestinal tissue samples (*n* = 28) for metagenomic and transcriptomic sequencing. The metagenomic data underwent microbial taxonomic structure analysis (including diversity and taxonomic composition) and microbial gene functional analysis (including KEGG Orthology and Cluster of Orthologous Groups analyses, and identification of CAZymes and antimicrobial resistance genes) to study the functional profiles along the GIT. The transcriptome data underwent gene expression analysis (with 13,793 genes expressed in intestinal tissues) and DEG analysis (with 1,618 genes identified as DEGs). Functional enrichment analysis was performed on the DEGs. Finally, correlation analysis was conducted among the 20 most abundant species in the SPF pig gut and the 130 most highly up-and down-regulated DEGs in the small intestine (after converting human genes to pig homologs) to understand differences in the gut microbiota and the microbial-host gene interactions in SPF pigs.

Three days before the expected delivery date, piglets were delivered through aseptic cesarean section. Six female piglets were randomly selected as subjects for this study (Figure 1). These piglets were reared following the SPF pig model construction and rearing standards (Zhang et al., 2023) on the standardized aseptic pig breeding platform at the Institute of Biotechnology, Chongqing Academy of Animal Science. In summary, SPF piglets were fed with sterilized milk powder (0–21 days) and pelleted feed (21–42 days) diluted 1:4 with sterile water and sterilized using Co60 γ radiation. During the feeding process, milk powder, feed, and water were introduced into the isolator through a transfer tube and disinfected with 1% peracetic acid to prevent microbial contamination.

After feeding for 42 days, in order to comprehensively study the gut microbial characteristics of SPF pigs and their interactions with the host, we collected a total of 42 GIT content samples and 28 intestinal tissue samples from 6 SPF Bama females at the Chongqing Academy of Animal Sciences. Among them, the GIT content samples included samples from four different sites, including 6 stomach samples, 18 small intestine samples (duodenum, jejunum, and ileum), 12 large intestine samples (cecum and colon), and 6 rectum samples. The intestinal tissue samples consisted of 17 small intestine samples (duodenum, jejunum, and ileum) and 11 large intestine samples (cecum and colon) (Supplementary Tables S1, S2). GIT contents and intestinal tissue samples from the SPF pigs were collected and stored at −80°C prior to sequencing.

### Community analysis of the GIT microbiome

Total DNA was extracted from GIT content samples using a QIAamp DNA Stool Mini Kit (Qiagen, Germany), following the manufacturer’s instructions. Each sample yielded 0.2 μg of total DNA, which was used as input material for DNA library preparation. Briefly, genomic DNA samples were fragmented by sonication to an average size of 350 bp. The DNA fragments then underwent end-polishing, A-tailing, and ligation with full-length adapters for Illumina sequencing, followed by PCR amplification. The PCR products were purified using the AMPure XP system from Beverly. Next, we assessed the quality of the libraries using the Agilent 5400 system and quantified by QPCR (1.5 nM). Based on the effective library concentration and the required amount of data, qualified libraries were pooled and sent to Novogene Bioinformatics Technology Co., Ltd. (Beijing, China) for metagenomic sequencing using the Illumina NovaSeq 6000 platform with a paired-end-150 bp strategy, aiming to generate at least 10 G of raw data per sample. Forty-two samples were sequenced, resulting in approximately 448.08 G of raw data. Basic quality processing was performed on the raw data using Fastp (Chen et al., 2018) (v0.23.1) to obtain clean data. The data processing steps were as follows: if either of the paired reads contained adapter contamination, the reads were discarded; if either of the paired reads had more than 10% of bases with uncertainty, the reads were discarded; if the proportion of low-quality bases (Phred quality < 5) in either of the paired reads exceeded 50%, the reads were discarded. To remove reads that might originate from the host or food, we downloaded the reference genome sequence of *Sus scrofa* (Sus_scrofa.Sscrofa11.1.dna.toplevel.fa) from Ensembl and aligned the sequencing data to the host genome using BWA-MEM (Li, 2013) (v0.7.17). Subsequently, we used SAMtools (Li et al., 2009) (v1.17) to remove host reads. After this step, we obtained approximately 271.94 G of filtered data and 1.866 billion high-quality paired-end filtered reads for subsequent analysis.

The high-quality reads for each sample were assembled using MEGAHIT (Li et al., 2015) (v1.2.9) with the parameter “-min-contig-len 500,” resulting in 446,890 contigs. The assembly quality was assessed using QUAST (Gurevich et al., 2013) (v5.2.0). Next, open reading frames were predicted using metaProdigal (Hyatt et al., 2010) (v2.6.3) with the parameter “-p meta,” resulting in 1,213,609 open reading frames from which 620,325 complete genes were extracted (51.1%, partial = 00; Supplementary Figure S1). Subsequently, CD-HIT (Huang et al., 2010) (v4.8.1) was employed to remove redundancy among the predicted genes and proteins. Clustering was performed using 95% sequence identity and 90% sequence coverage, resulting in the formation of the most similar clusters. The nucleotide sequences were then translated into their corresponding protein sequences. The abundance of non-redundant genes was calculated as transcripts per million (TPM) fragments using Salmon (Patro et al., 2017) (v1.10.1).

To obtain the taxonomic composition of the gut microbiota for each sample, we used Kraken 2 (Lu et al., 2022) (v2.1.3) with the default parameters to align the translated protein sequences against its database (k2_pluspf_20231009). We calculated alpha diversity based on the Shannon index using the vegen package in R (v4.3.1), computed beta diversity distances using Bray–Curtis dissimilarity, and performed species rarefaction analysis using USEARCH (Edgar, 2010) (v10.0) (Figure 1). Differences in the GIT microbiota between SPF pigs were assessed using the Wilcoxon test. Spearman rank correlation was used to calculate the correlation coefficients of the 20 most abundant microorganisms at the species level in the GIT. Only correlations with Spearman correlation coefficient (r) ≥ 0.3 or ≤ −0.3 and *p* < 0.05 were retained and visualized using R (v4.3.1). Linear discriminant analysis implemented in the LEfSe (Segata et al., 2011) tool was used to identify different taxonomic groups between sample groups. We identified discriminative groups with a linear discriminant analysis score > 3 and assessed the statistical significance of abundance differences between GIT microbiota groups in SPF pigs using the Wilcoxon test.

### Functional analysis of the GIT microbiome

We used GhostKOALA (Kanehisa et al., 2016) to map non-redundant protein sequences to the KEGG database for KEGG Orthology annotation. For protein Cluster of Orthologous Groups annotation, we aligned non-redundant protein sequences to the eggNOG database (v5.0.2) using the default parameters of eggNOG-mapper (Cantalapiedra et al., 2021) (v2.1.12). Additionally, we used the BLASTp option of DIAMOND (Buchfink et al., 2015) (v2.0.15) with the parameter “-e 1e-102” to align non-redundant protein sequences to the latest CAZy database (CAZyDB.07262023) for the detection of carbohydrate-active enzymes (CAZymes). To detect antimicrobial resistance genes in each microorganism, we used the RGI (Alcock et al., 2023) (v5.2.0) tool with the option “DIAMOND --include_loose” to align non-redundant protein sequences to the latest CARD database (v3.2.6) (Figure 1).

### Transcriptome analysis of intestinal tissues

We extracted total RNA from intestinal samples using the Trizol method. After qualifying the RNA samples, eukaryotic mRNAs were enriched using Oligo(dT) magnetic beads. For prokaryotes, a mRNA purification kit to remove rRNA and enrich mRNA was used. Subsequently, we added a cleavage buffer to break the mRNA into short fragments, which was then used as a template to synthesize single-stranded cDNA using random hexamers. Next, a buffer, dNTPs, DNA polymerase I, and RNase H were added to synthesize double-stranded cDNA, which was then purified using AMPure XP beads. The cDNA then underwent end repair, A-tailing, and sequencing adapter ligation, followed by fragment size selection using AMPure XP beads. Finally, PCR amplification was performed, and the PCR products were purified using AMPure XP beads to obtain the final library. After construction, we quantified the library using Qubit 2.0 and diluted it accordingly. Subsequently, we used the Agilent 2100 system to assess the insert size of the library, ensuring that the insert fragments met the expected size. Next, we used q-PCR to accurately quantify the effective concentration of the library, ensuring library quality. After passing the quality check, the library was sequenced using DNBSEQ-T7.

The data obtained from sequencing is referred to as raw reads. Prior to reference genome alignment, we obtained clean reads by filtering out reads containing adapters (adapter contamination), reads containing unknown bases (N bases), and reads with low sequencing quality. Based on the pig reference genome and annotation files downloaded from Ensembl, we performed alignment of the clean reads using STAR (Dobin et al., 2012) (v2.7.10b), assembly using Cufflinks (Trapnell et al., 2010) (v2.2.1) and TACO (Niknafs et al., 2017) (v0.7.3), and calculation of the protein-coding potential of the assembled transcripts using CPC (Kang et al., 2017) (v2.0). Ultimately, we obtained reference genome files for mRNAs and lncRNAs, which were used for subsequent analysis. We quantified the data using Kallisto (Bray et al., 2016) (v0.49.0) to obtain the counts and TPM values for mRNAs and lncRNAs, which were used for downstream analysis. After calculating expression levels, we grouped the samples based on treatments and conditions. First, we selected expressed genes by choosing genes that were expressed (TPM > 0) in all samples. Subsequently, differential gene expression analysis was conducted using the edgeR package (Chen et al., 2016) in R (v4.3.1). We defined differentially expressed genes (DEGs) as genes with |Log_2_(FoldChange)| > 1 and FDR.P (false discovery rate adjusted *p*-value) < 0.05. Kyoto Encyclopedia of Genes and Genomes (KEGG) (Kanehisa and Goto, 2000) and Gene Ontology (GO) (Gene Ontology Consortium, 2007) enrichment analyses were performed using the clusterProfiler package (Wu et al., 2021) (Figure 1). The gene sets used were primarily of mouse or human origin; therefore, we retained only the genes that exhibited one-to-one orthology with pigs.

### Comprehensive analysis of the interaction between the host transcriptome and the gut microbiota

To investigate potential functional interactions between DEGs in the host gut and the gut microbiota (top 20 species), we performed Spearman rank correlation analysis. We defined DEGs as genes with |Log_2_(FoldChange)| > 1 and FDR.P < 0.05. Specifically, we selected the 130 most up-and down-regulated DEGs based on |Log_2_(FoldChange)| (after converting human genes to pig homologs). Spearman rank correlation, *p*-values and significant associations (FDR.P < 0.05) between genes were calculated using the corr.test() function from the psych package in R.

## Results

### Taxonomic characteristics of microbiota at different GIT sites

**Figure 2:**
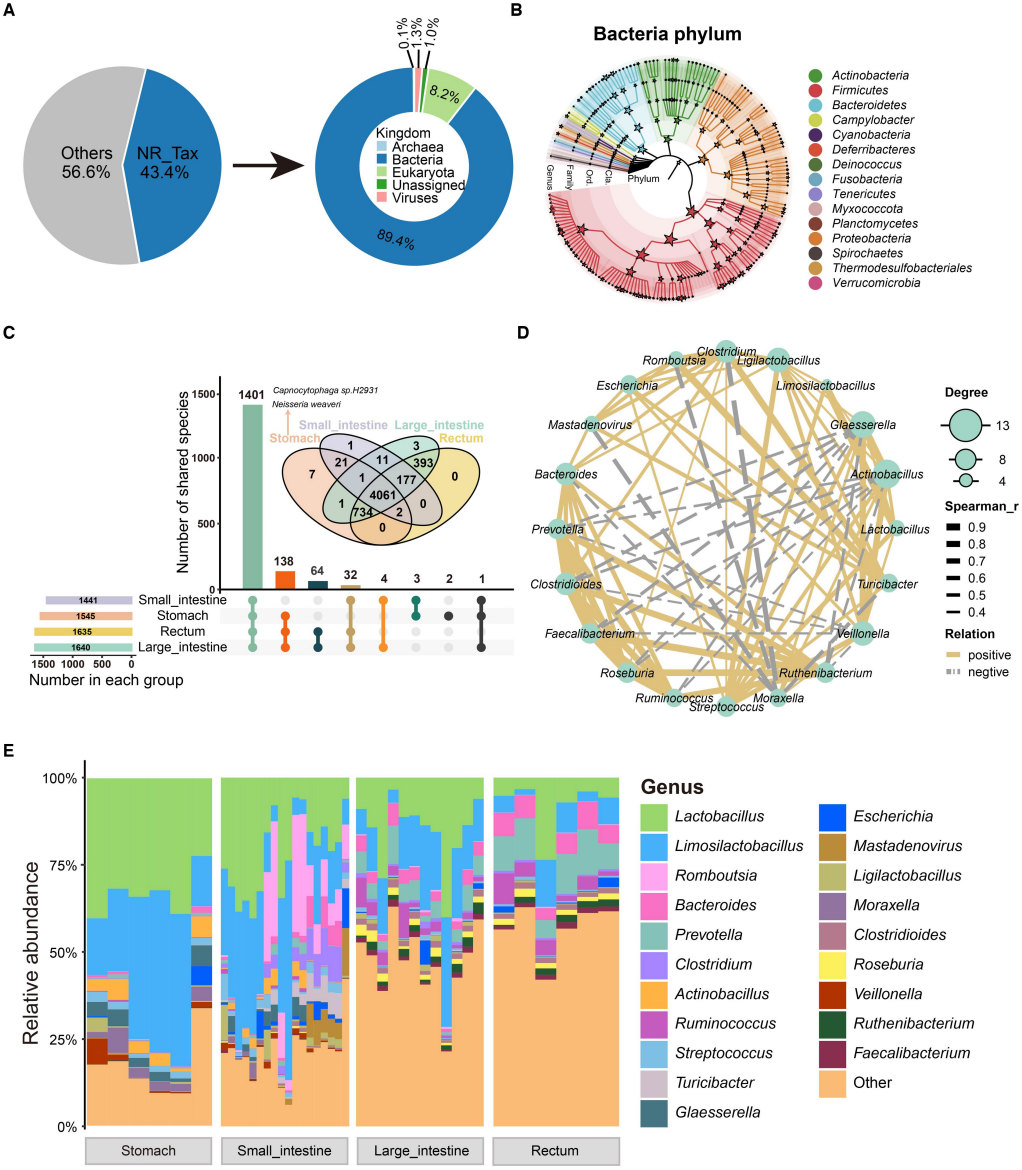
Taxonomic characteristics and co-occurrence network of GIT microbiota in SPF pigs. **(A)** Taxonomic annotation of microbial proteins in SPF pigs. The pie chart shows the proportion of annotated proteins (left) and the overall composition of the microbiota at the kingdom level (right). **(B)** The phylogenetic relationships and taxonomic classification of 146 bacterial genera (having >150 mapped reads) in the SPF pig GIT were constructed using GraPhlAn. Stars on internal branches and leaf branches represent branches at each taxonomic level; coloring is based on phylum level branches. **(C)** Shared and unique relationships of GIT microbiota in SPF pigs at different taxonomic levels. Features are considered to be present in a group only if the total abundance of samples in that group is greater than 0. The UpSet plot shows shared and unique microbiota at the genus level in the GIT of SPF pigs, while the Venn diagram above shows shared and unique microbiota at the species level. **(D)** Co-occurrence network analysis of microbial genera based on the 20 most abundant genera in the GIT of SPF pigs. Solid lines indicate Spearman rank correlation coefficient > 0.30; dashed lines indicate Spearman rank correlation coefficient < −0.30. Node size is proportional to the degree (the number of connections a node has with other nodes). **(E)** Genus-level composition of microbiota at different sites along the GIT of SPF pigs. X-axis: different sites (stomach, small intestine, large intestine, and rectum), Y-axis: relative abundance of microbiota.

We used the species annotation software, Kraken2, to classify the non-redundant protein sequences from the GIT of SPF pigs. A total of 269,251 non-redundant proteins (43.4% of total proteins) were annotated into four domains (Bacteria 89.4%, Eukaryota 8.2%, Viruses 1.3%, and Archaea 0.1%; Figure 2A). Sixty-one phyla, 113 classes, 226 orders, 470 families, 1,646 genera, and 5,421 species were annotated (Supplementary Table S3). We then performed rarefaction analysis on the 5,421 annotated species. Moreover, with increasing sequencing depth, the species rarefaction curves obtained from different GIT sites reached a saturation stage (Supplementary Figure S2A).

We then performed statistical analysis on the dominant phyla and genera in the GIT. The dominant phyla were *Firmicutes*, *Proteobacteria*, and *Bacteroidetes*, while the dominant genera were *Lactobacillus*, *Limosilactobacillus*, and *Romboutsia* (Supplementary Figure S2B). Additionally, to demonstrate better, we constructed an evolutionary tree using the selected bacteria after filtering and annotated them to 15 phyla and 146 genera. The four most abundant bacterial phyla (dominant phyla) were *Firmicutes*, *Proteobacteria*, *Actinobacteria*, and *Bacteroidetes* (Figure 2B), which is consistent with studies conducted on humans and other animals (Xiao et al., 2017; Long et al., 2022; Maiuolo et al., 2022; Yu et al., 2023). To further investigate changes in the relative abundance of microbiota along the GIT, we analyzed the genus-level microbial composition, as shown in Figure 2E. We found that *Lactobacillus*, *Limosilactobacillus*, *Romboutsia*, and *Bacteroides* were the predominant bacteria in the GIT. These bacteria are considered probiotics and play a significant role in maintaining GIT health of animals (Abuqwider et al., 2022). Both *Lactobacillus* and *Limosilactobacillus* exhibited a gradual decrease in abundance moving anally along the GIT (Figure 2E).

Using 0 as the threshold for presence (a feature is considered present in a group only if the total abundance across all samples in that group is greater than the threshold), we found that 1,441, 1,545, 1,635, and 1,640 genera were present in samples from the four GIT sites (stomach, small intestine, large intestine, rectum) (Figure 2C). Additionally, we observed that 1,401 genera (85% of all annotated genera) were present in all four GIT sites, 138 genera (8%) were present in samples from three sites, and only a small fraction of genera showed site-specificity (two in the stomach; Figure 2C). According to these criteria, we did not find any site-specific genera in other sites, possibly because of the similar composition of microbial species along the SPF pig GIT. At the species level, seven showed gastric specificity. Among them, the most abundant were *Capnocytophaga* sp. *H2931* [SUM Abundance (all samples) = 543.11] from the *Capnocytophaga* genus [a Gram-negative bacterial genus typically found in the oral-pharyngeal cavity of mammals and associated with the pathogenesis of periodontal disease and infection following animal bites (Jolivet-Gougeon et al., 2007)], and *Neisseria weaveri* [SUM Abundance (all samples) = 395.36] from the *Neisseria* genus [a Gram-negative bacterial genus colonizing the mucosal surfaces of various animals (Panagea et al., 2002)] (Figure 2C). The reason for their high abundance might result from their advantageous use of nutrients compared with other gastric-specific microorganisms. Alternatively, they may have a close interaction with receptors on the gastric mucosa that interferes with the colonization of other microbes.

We analyzed microbial diversity further and found significant differences in alpha diversity among all GIT sites based on the Shannon index measurement (pairwise Wilcoxon rank-sum test, *p* < 0.05; Supplementary Figure S3A). The rectum exhibited the highest diversity, followed by the large intestine and small intestine, while the stomach showed the lowest diversity (Supplementary Figure S3A). However, considering different sources, the duodenum displayed the lowest diversity (Supplementary Figure S3B). To further determine the overall microbial characteristics of the four GIT sites, beta diversity comparisons were performed using paired non-parametric multivariate analysis of variance.

Based on principal coordinate analysis (PCoA) of Bray-Curtis distances between samples, the difference between the four GIT sites was extremely significant (*R*^2^ = 0.4441, *p* = 0.001, paired non-parametric multivariate analysis of variance; Supplementary Figure S3C). Interestingly, samples from the cecum, colon, and rectum formed a highly clustered group (Supplementary Figure S3D), indicating strikingly similar microbial community structures in these three sources. This is consistent with their alpha diversity, as there was little difference in alpha diversity among these three sources (Supplementary Figure S3B).

To investigate the interactions among the GIT microbiota of SPF pigs, we selected core genera (average relative abundance >0.5%) and calculated the Spearman rank correlation coefficients, as shown in Figure 2D. *Lactobacillus* and *Limosilactobacillus* (the two most abundant genera) exhibited a strong positive correlation (Spearman’s rank correlation coefficient = 0.95, *p* = 0), indicating a potential mutualistic relationship between them. Similarly, there was a strong positive correlation among the three genera with the highest degree of centrality (*Actinobacillus*, *Glaesserella*, and *Veillonella*) (Figure 2D). In contrast, *Moraxella* showed a strong negative correlation with *Clostridium*, *Romboutsia*, and *Mastadenovirus*, indicating a potential competitive inhibition effect among them. Additionally, certain beneficial gut bacteria, such as *Prevotella*, *Clostridioides*, *Faecalibacterium*, *Roseburia*, *Ruminococcus*, and *Ruthenibacterium*, exhibited positive correlations with each other, forming a relatively independent and stable cluster (Figure 2D). Overall, the richness and diversity of the SPF pig GIT microbiota gradually increased from the stomach to the rectum. Moreover, these microorganisms formed a stable symbiotic network within the GIT.

In the ileum, we identified the presence of *Porcine adenovirus A*, which belongs to the phylum *Preplasmiviricota* (Supplementary Figure S3E). Infection with this virus is usually subclinical in pigs, and when clinical symptoms do occur, they manifest as mild and transient GIT symptoms, such as diarrhea, anorexia, and dehydration, and are most commonly observed in piglets (Liu et al., 2020; Gainor et al., 2022).

### Different bacteria along the GIT correspond to GIT functions

**Figure 3:**
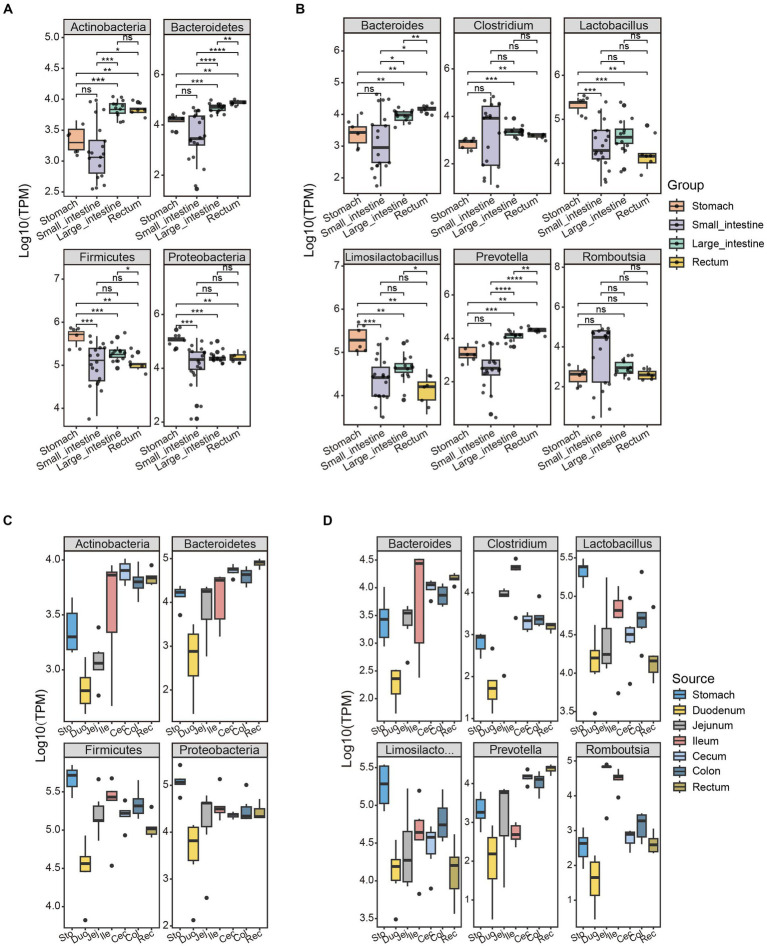
Unique patterns of bacterial abundance in the GIT of SPF pigs are associated with physiological functions. The abundance distribution patterns of the four bacterial phyla with the highest abundance in the GIT of SPF pigs were selected along the **(A)** Group and **(C)** Source. The abundance distribution patterns of the six bacterial genera with the highest abundance in the GIT of SPF pigs were selected along the **(B)** Group and **(D)** Source. Y-axis: TPM values transformed by logarithm base 10. The different colors of the boxes represent different parts of the GIT: stomach (orange, *n* = 6), small intestine (purple, *n* = 18), large intestine (green, *n* = 12), and rectum (yellow, *n* = 6). Pairwise Wilcoxon rank-sum tests were used to compare the groups. The boxplots display the median, 25th and 75th percentiles; solid lines represent the minimum and maximum values, and points falling outside the whiskers of the boxplot represent outliers. Significance levels: ^ns^*p* ≥ 0.05, *p < 0.05, **p < 0.01, ***p < 0.001, ****p < 0.0001.

The overall microbial structures in the stomach, small intestine, large intestine, and rectum differ from one another. The phyla, *Firmicutes* and *Bacteroidetes*, were the two most abundant phyla, accounting for 95% (stomach), 86% (small intestine), 71% (large intestine), and 55% (rectum) of the relative bacterial abundance in the four GIT sites (Supplementary Table S4). Interestingly, the *Actinobacteria* and *Bacteroidetes* phyla showed a increasing trend in abundance moving anally along the GIT, while the *Firmicutes* and *Proteobacteria* phyla exhibited the opposite pattern (Figures 3A,C). Based on this pattern, the ratio of *Firmicutes* to *Bacteroidetes* (F/B) was lowest in the rectum and highest in the stomach. The F/B ratio is associated with energy harvesting (obesity) (Ley et al., 2006; Turnbaugh et al., 2006); therefore, its trend aligns with the physiological changes along the GIT, from food digestion (in the stomach) to energy harvesting (in the intestine).

The abundance of the phylum *Bacteroidetes* did not differ significantly between the stomach and small intestine (pairwise Wilcoxon rank-sum test, *p* > 0.05), but did show significant differences in abundance among other GIT sites (Figures 3A,C). This may be because of the presence of gastric acid, bile, and the lower pH in the stomach and small intestine affecting the survival and proliferation of the *Bacteroidetes* (Ding and Shah, 2007). Several functions have been reported for *Bacteroidetes* in the GIT. These microorganisms are involved in the fermentation of carbohydrates, converting complex carbohydrates into usable products (Hu et al., 2020; Fan and Pedersen, 2021). Additionally, they are capable of using nitrogenous substances and participating in the biotransformation of bile acids and other sterols in metabolic processes (Guzior and Quinn, 2021).

*Firmicutes* was the most abundant phylum in the stomach because of the high abundance of the genera *Lactobacillus* and *Limosilactobacillus* (Figure 3B). Microorganisms belonging to these genera are able to survive and establish themselves in the highly acidic environment of the stomach (Su et al., 2011). *Lactobacillus* is a common beneficial bacterium and is often used as a probiotic in yogurt and other fermented dairy products (Oleksy and Klewicka, 2018). These microorganisms can produce acetate (a beneficial short-chain fatty acid), lactate, and antimicrobial substances, which help to inhibit pathogens (Tomova et al., 2019). A higher ratio of *Firmicutes* to *Bacteroidetes* in the gut of obese subjects has been observed, which may be associated with more efficient absorption of calories from food by *Firmicutes*, leading to obesity (Moreira Júnior et al., 2021) One reason for the increase in abundance of the phylum *Bacteroidetes* in the GIT is the higher abundance of the genus *Prevotella*. *Prevotella* is a major genus within the *Bacteroidetes*, with a high abundance in the rectum and significantly lower abundance in other sites (Figures 3B,D). *Prevotella* is associated with the degradation of non-fibrous plant tissue and can produce short-chain fatty acids through the fermentation of cellulose and other non-digestible carbohydrates (Rubino et al., 2017). Additionally, *Prevotella* plays an important role in influencing host immune regulation (Iljazovic et al., 2021).

There was a significant difference in the abundance of the genus *Clostridium* within the phylum *Firmicutes* between the stomach and the large intestine/rectum (pairwise Wilcoxon rank-sum test, *p* < 0.05; Figure 3B). This may be because of notable differences in environmental conditions between the stomach and the large intestine/rectum (Sengupta et al., 2014; Chu and Traverso, 2022) The stomach is an acidic environment with a high concentration of gastric acid, which has inhibitory effects on many bacteria. Certain strains within the genus *Clostridium* may have weaker resistance to the acidic environment, resulting in lower numbers in the stomach. In contrast, the environment of the large intestine and rectum is neutral to slightly alkaline, providing more suitable growth conditions for *Clostridium* strains, allowing them to thrive and reproduce. However, there was no significant difference in the abundance of the genus *Romboutsia* across different sites of the GIT (pairwise Wilcoxon rank-sum test, *p* > 0.05; Figure 3B). This may be because strains within the genus *Romboutsia* have relatively stronger adaptability to gastric acid and intestinal environments, allowing them to survive and reproduce in different locations. The varying abundance of these bacterial genera reflect the interactive trends between the microbiota and the different GIT sites that collectively promote digestion and absorption functions along the GIT.

Additionally, we employed a linear discriminant analysis effect size (LEfSe) and linear discriminant analysis scores >3 to identify 52 taxonomic groups with significant differences in abundance at the genus level (Supplementary Figure S4). Among these taxonomic groups, the genera *Lactobacillus*, *Romboutsia*, *Roseburia*, and *Prevotella* were significantly more abundant in the stomach, small intestine, large intestine, and rectum compared with other taxonomic groups (Supplementary Figure S4). Other taxonomic groups with significant differences in abundance may also contribute to the physiological functions of the corresponding GIT sites. Therefore, further research is needed to explore their specific roles.

### Functional characteristics of microbiota in different sites of the GIT

**Figure 4:**
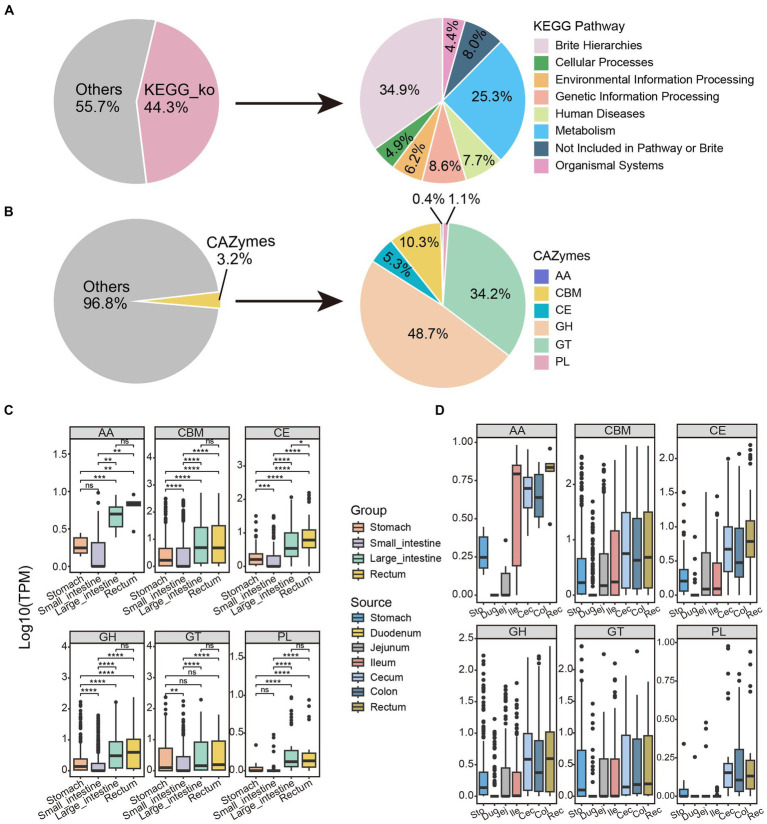
Functional annotation and distribution of non-redundant proteins along the GIT in SPF pigs. Functional annotation of microbial protein sequences in SPF pigs was performed using **(A)** GhostKOALA and **(B)** dbCAN2. The pie charts display the proportions of proteins annotated by these two methods (left) and the overall categories (right). The box plots illustrate the abundance distribution patterns of the six major families of CAZymes along the **(C)** Group and **(D)** Source. AA, auxiliary activities; CBM, carbohydrate-binding modules; CE, carbohydrate esterases; GH, glycoside hydrolases; GT, glycosyltransferases; PL, polysaccharide lyases. Y-axis: TPM values transformed by logarithm base 10. Boxes of different colors represent different segments of the GIT: stomach (orange, *n* = 6), small intestine (purple, *n* = 18), large intestine (green, *n* = 12), and rectum (yellow, *n* = 6). Pairwise Wilcoxon rank-sum tests were used to compare the groups. The box plots display the median, 25th, and 75th percentiles, while the solid lines represent the minimum and maximum values. Points outside the whiskers of the box plots indicate outliers. Significance levels are denoted as follows: ^ns^*p* ≥ 0.05, *p < 0.05, **p < 0.01, ***p < 0.001, and ****p < 0.0001.

As shown in Figure 4A, nearly half of the proteins (274,379, 44.3% of the total) were annotated to KEGG Orthology pathways. KEGG pathway mapping indicated the most abundant pathways in the GIT of SPF pigs to be metabolic pathways (25.3%). Additionally, a significant proportion of the proteins (34.9%) could be assigned to BRITE hierarchical classifications (Figure 4A). According to KEGG functional analysis (Supplementary Figure S5), the microbial community in the small intestine exhibited richness in nucleotide metabolism, xenobiotic biodegradation, and metabolism, as well as digestive system. The microbial community in the large intestine was enriched in functions related to amino acid metabolism, biosynthesis of other secondary metabolites, and energy metabolism. Interestingly, similar results were obtained based on eggNOG analysis (Supplementary Figures S6A,E). These findings are consistent with the large intestine being a site of abundant microbial fermentation and the production of various metabolites, such as amino acids and short-chain fatty acids (Williams et al., 2001; Dai et al., 2011; Vital et al., 2014; Oliphant and Allen-Vercoe, 2019), which are crucial for host health. Therefore, we believe that the differences in composition and functionality of the microbial communities in the small and large intestines are related to their positions and nutritional conditions in the digestive system.

According to the CAZy database, only a small fraction of proteins (19,857, 3.2% of the total) were annotated as CAZymes (Figure 4B). Similar to studies in ruminant animals (Tong et al., 2022; Cao et al., 2023), the majority of these enzymes were annotated as glycoside hydrolases (9,664, 48.7% of 19,857), followed by glycosyltransferases (6,788, 34.2%) and carbohydrate-binding modules (2,052, 10.3%). The other three classes of enzymes, including carbohydrate esterases (1,048, 5.3%), polysaccharide lyases (221, 1.1%), and auxiliary activity (74, 0.4%), were represented by much smaller numbers of proteins.

We calculated the abundance of all proteins in each sample and compared their distribution. As shown in Figure 4C, the abundance of six CAZy families was relatively similar in the large intestine and rectum, but was significantly different from the other two sites (pairwise Wilcoxon rank-sum test, *p* < 0.05). Interestingly, the abundance of all CAZy protein families showed a trend of initially decreasing and then increasing moving anally along the GIT, with the lowest abundance in the small intestine and the highest in the rectum (Figure 4C). This might be because the small intestine is primarily responsible for nutrient absorption (Chin et al., 2017), not carbohydrate breakdown, resulting in a lower demand for CAZymes. In contrast, the microbial diversity was significantly increased in the rectum (Supplementary Figure S3A), where abundant CAZymes are involved in the degradation and fermentation of complex carbohydrates. Therefore, this trend reflects the characteristics and changing demands of different GIT sites in carbohydrate digestion and metabolism.

There were no significant differences in abundance of polysaccharide lyases and auxiliary activity enzymes between the stomach and small intestine, while differences in carbohydrate-binding modules, carbohydrate esterases, glycoside hydrolases, and glycosyltransferases were highly significant (pairwise Wilcoxon rank-sum test, *p* < 0.05; Figure 4C). This may be because polysaccharide lyases and auxiliary activity enzymes are primarily involved in the degradation of polysaccharides, while carbohydrate-binding modules, carbohydrate esterases, glycoside hydrolases, and glycosyltransferase enzymes have different functions, such as polysaccharide structure recognition, binding, and modification (Ndeh et al., 2017; Ye et al., 2020). Therefore, the degradation and digestion processes of polysaccharides were relatively similar in the stomach and small intestine. Interestingly, unlike other families, the abundance differences of glycosyltransferases in the stomach, large intestine, and rectum were not significant (Figure 4C; pairwise Wilcoxon rank-sum test, *p* > 0.05). This can be attributed to similar expression patterns and functions of glycosyltransferases in these tissues and indicates that they might be expressed at similar levels in the stomach, large intestine, and rectum, resulting in non-significant differences in their abundances. The duodenum had the lowest abundance among the six families (Figure 4D); therefore, we identified it as an outlier, possibly because of its lowest alpha diversity (Supplementary Figure S3B).

We investigated the distribution of CAZyme subfamilies in four GIT sites. The most abundant subfamily in the GIT was carbohydrate-binding module (CBM)48 (Supplementary Figure S6D), which possesses the ability to recognize and bind polysaccharide substrates, particularly xylan substances (Zhang et al., 2022). Interestingly, CBM91 exhibited lower abundance in the small intestine and stomach, but higher abundance in the large intestine and rectum (Supplementary Figure S6D). CBM91 is capable of binding xylan substances (such as birch and oat bran) (Ito et al., 2022), and the large intestine and rectum are the sites of fiber digestion (Moran and Bedford, 2022). The abundance of CBM50 gradually decreased along the GIT (Supplementary Figure S6D); CBM50 is involved in the degradation of chitin or peptidoglycans (Gruber et al., 2011). CBM6 exhibited higher abundance in the large intestine and rectum. CBM34, which binds granular starch (Kuchtová and Janeček, 2016), showed higher abundance in the stomach and small intestine, but the lowest abundance in the rectum (Supplementary Figure S6D). These differences may be closely associated with the host’s diet.

We also performed protein sequence alignment with the Comprehensive Antibiotic Resistance Database (CARD) and found a total of 43,892 protein sequences that matched antibiotic resistance genes (accounting for 7.1% of the total; Supplementary Figure S6B). The most abundant protein in the GIT was *macB* (Supplementary Figure S6C), which forms an antibiotic efflux complex with *macA* and *TolC*. The resistance mechanism of this complex is achieved through antibiotic efflux, with the best antibody match being against *Neisseria gonorrhoeae* (Xu et al., 2009). It is worth noting that the abundance of *vanU gene in vanG cluster* and *evgS* were higher in the large intestine and rectum (Supplementary Figure S6C) and lower in the stomach and small intestine. Their resistance mechanisms involve antibiotic target alteration and antibiotic efflux, with the best matches to antibodies against *Streptococcus agalactiae* and *Escherichia coli* (Nishino and Yamaguchi, 2002; Boyd et al., 2006). The highest abundance of *RanA* was found in the stomach and small intestine, while it was lower in the large intestine and rectum (Supplementary Figure S6C). *RanA*, together with *RanB*, confers resistance to aminoglycoside antibiotics through antibiotic efflux, with the best to the antibody match being against *Riemerella anatipestifer* (Li et al., 2020). Therefore, we believe there are different antimicrobial resistance genes present in different sites of the SPF pig GIT that have varying resistance mechanisms and best antibody matches. The identification of abundance differences of these antimicrobial resistance genes in the GIT provides background information to inform antibiotic use in pig farming. In summary, these findings reveal the microbial composition and functional differences within the four GIT sites of SPF pigs, along with characteristics associated with digestion and metabolism.

### Transcriptome differences between the small intestine and large intestine

We determined transcriptome differences between the small intestine (*n* = 17) and large intestine (*n* = 11) by sequencing the transcriptomes of 28 tissue samples. In conjunction with the metagenomic data described above, we explored the interaction between SPF pig intestinal microbiota and the host.

**Figure 5:**
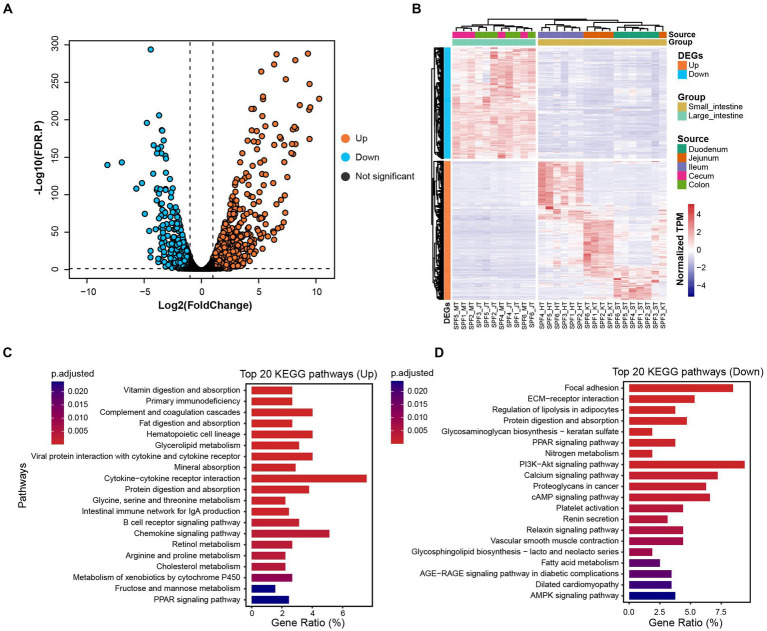
Distribution of DEGs and their associated functional enrichment in small and large intestines of SPF pigs. **(A)** Volcano plot of DEGs between the small intestine (*n* = 17) and large intestine (*n* = 11). DEGs with |Log_2_(FoldChange)| > 1 and FDR. *p* < 0.05 are colored orange (up-regulated in the small intestine) and blue (down-regulated in the small intestine). **(B)** Composition of DEGs between the small intestine (yellow) and large intestine (green). **(C)** Top 20 KEGG enrichment pathways for up-regulated genes in the small intestine (adjusted *p* < 0.05). **(D)** Top 20 KEGG enrichment pathways for down-regulated genes in the small intestine (adjusted *p* < 0.05). X-axis: gene ratio (number of genes enriched in the pathway/total number of genes enriched in all pathways).

By comparing gene expression profiles from different sites of the host GIT, we identified 1,618 DEGs between the small and large intestines (|Log_2_(FoldChange)| > 1 and FDR.P < 0.05). Among these genes, 912 were up-regulated and 706 were down-regulated in the small intestine (Figures 5A,B).

We observed significant enrichment of up-regulated genes in multiple KEGG pathways in the small intestine (Figure 5C). Specifically, these up-regulated genes were significantly enriched in digestion and absorption-related pathways, including vitamin digestion and absorption, fat digestion and absorption, mineral absorption, and protein digestion and absorption (adjusted *p* < 0.05). Furthermore, immune-related pathways, such as primary immunodeficiency, complement and coagulation cascades, hematopoietic cell lineage, and viral protein interaction with cytokine and cytokine receptor, also showed significant enrichment (adjusted *p* < 0.05). We also observed significant enrichment in metabolism-related pathways, including glycerolipid metabolism, glycine, serine, and threonine metabolism, retinol metabolism, and arginine and proline metabolism (adjusted *p* < 0.05). Therefore, we conclude that the main functions of the SPF pig small intestine involve digestion and absorption, metabolism, and immune responses.

For the down-regulated genes in the small intestine (Figure 5D), we found significant enrichment in signaling transduction-related pathways, including focal adhesion, ECM-receptor interaction, and the PPAR signaling pathway (adjusted *p* < 0.05). Additionally, significant enrichment was observed in pathways related to nutrient metabolism, such as regulation of lipolysis in adipocytes, protein digestion and absorption, glycosaminoglycan biosynthesis—keratan sulfate, and nitrogen metabolism (adjusted *p* < 0.05). Therefore, we conclude that the main functions of the SPF pig large intestine involve nutrient metabolism and disease-related processes.

Further analysis of the GO enrichment results for Biological Process (BP) terms (Supplementary Figure S7A) revealed that the up-regulated genes in the small intestine were primarily enriched in immune system-related terms, such as mononuclear cell proliferation, leukocyte proliferation, lymphocyte proliferation, and lymphocyte differentiation (adjusted *p* < 0.05). Additionally, significant enrichment was observed in terms related to metabolic processes, such as alcohol metabolic process, and organic anion transport (adjusted *p* < 0.05). This indicates that the small intestine plays an important role in substance transport, homeostatic regulation, immune modulation, and cell activation.

The enriched down-regulated genes in the small intestine (Supplementary Figure S7B), were mainly enriched in terms related to extracellular matrix organization and development, such as extracellular matrix organization, extracellular structural organization, external encapsulating structure organization, wound healing, and cell-substrate adhesion (adjusted *p* < 0.05). Terms related to metabolic regulation, such as fatty acid metabolic process, and response to steroid hormone (adjusted *p* < 0.05), were also enriched. This indicates that the large intestine primarily functions in extracellular matrix organization and development, as well as nutrient metabolism.

In summary, there are differences in KEGG and GO enrichment between the small and large intestines, reflecting their specific biological functions and physiological processes. The small intestine is mainly involved in processes related to digestion and absorption, metabolism, and immune processes, while the large intestine is more involved in processes related to nutrient metabolism. These differences are consistent with our previous findings on the functional differences between the microbiota of the small and large intestines (Supplementary Figure S5). Overall, these findings indicate a co-evolutionary trend between the gut microbiota of SPF pigs and their hosts. These results provide important clues and foundations for further research on the characteristics and functions of SPF pig intestinal tissues, and contribute to a better understanding of the biological functions of the intestine and its role in host health.

### Coevolution of host DEGs and gut microbiota

**Figure 6:**
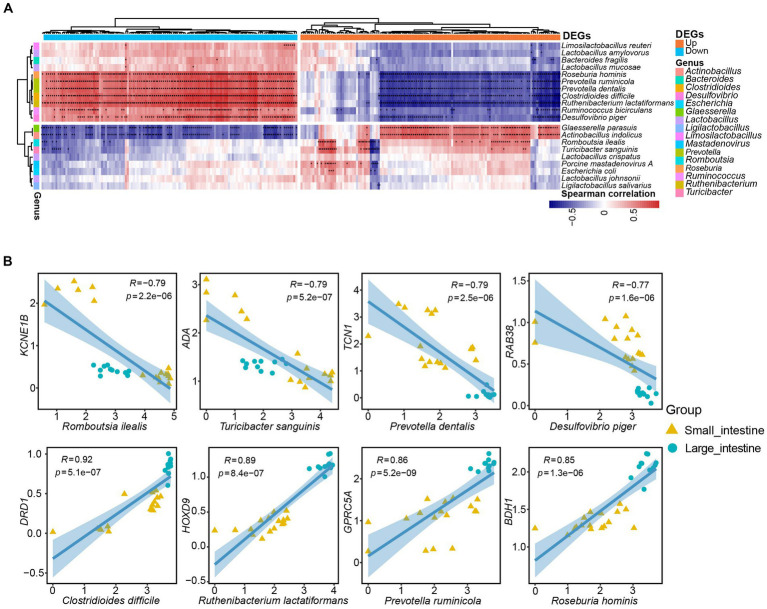
Specific strong associations between SPF pig gut microbiota and host genes. **(A)** Correlation analysis was performed between the top 20 ranked species-level microbiota in the SPF pig gut and the top 130 differentially expressed genes (DEGs) in the small intestine with |Log_2_(FoldChange)| (a total of 260 DEGs after converting human genes to pig homologs). The horizontal bars in orange and blue represent up-regulated and down-regulated genes in the small intestine, respectively, while the 16 vertical bars of different colors represent genera corresponding to the species-level microbiota. Only Spearman rank correlations with *p*-value < 0.01 were considered as strong associations and are marked with “*” in the heatmap (*p < 0.01, *p* > 0.001, and **p < 0.001). The red and blue cells in the heatmap indicate positive and negative correlations between genes and microbiota, respectively. **(B)** Scatter plots depict the grouping patterns of the eight pairs of gene-microbiota correlations of interest in the small intestine (yellow, *n* = 17) and large intestine (green, *n* = 11) samples. X and Y axes: TPM values transformed by logarithm base 10. Spearman rank correlation (R) and significance (*p*) are displayed at the top of each graph.

To investigate correlations between host genes and the gut microbiota and potential roles that any associations may have in intestinal function, we conducted a correlation analysis between 260 DEGs and the 20 most abundant microbial taxa in the SPF pig gut. We identified 1729 specific and strong microbiota-host associations in the small intestine and large intestine (Spearman rank correlation ≥ 0.5 or ≤ −0.5, FDR.P ≤ 0.01). Interestingly, the microbial taxa associated with up-regulated and down-regulated DEGs in the small intestine were mostly distinct (Figure 6A). Specifically, we identified 11 microbial taxa (*Limosilactobacillus reuteri*, *Lactobacillus amylovorus*, *Bacteroides fragilis*, *Lactobacillus mucosae*, *Roseburia hominis*, *Prevotella ruminicola*, *Prevotella dentalis*, *Clostridioides difficile*, *Ruthenibacterium lactatiformans*, *Ruminococcus bicirculans*, and *Desulfovibrio piger*) that showed positive correlations with most down-regulated genes but negative correlations with up-regulated genes (Figure 6A). In contrast, we found that nine microbial taxa (*Glaesserella parasuis*, *Actinobacillus indolicus*, *Romboutsia ilealis*, *Turicibacter sanguinis*, *Lactobacillus crispatus*, *Porcine mastadenovirus A*, *Escherichia coli*, *Lactobacillus johnsonii*, and *Ligilactobacillus salivarius*) exhibited positive correlations with the majority of up-regulated genes but negative correlations with down-regulated genes (Figure 6A).

We then selected eight microbial taxa of interest to describe the pairwise correlations between host gene expression and microbial taxonomic groups (Figure 6B). The *TCN1* gene encodes vitamin B12-binding protein, which binds to and protects vitamin B12 from the acidic environment of the stomach (Johnston et al., 1992). There was a strong negative correlation between *Prevotella dentalis* and *TCN1* gene expression (*R* = −0.79, *p* = 2.5e-06; Figure 6B). This negative correlation may be attributed to *Prevotella dentalis* being a Gram-negative bacteria with metabolic pathways related to vitamin B12 (Franco-Lopez et al., 2020). The transport protein encoded by the *TCN1* gene is responsible for transporting vitamin B12 into cells and participating in the metabolic processes in which it is involved. If *Prevotella dentalis* is involved in the intracellular metabolism of vitamin B12, a negative correlation with *TCN1* expression may exist. Similarly, the *ADA* gene encodes adenosine deaminase, an enzyme involved in adenosine metabolism. Adenosine is an important cell signaling molecule that plays a significant role in regulating intestinal immune function and inflammatory responses. The function of adenosine deaminase is to convert adenosine into inosine, thereby regulating adenosine levels (Eltzschig et al., 2006). Although the exact function and impact of *Turicibacter sanguinis* in the intestines are still under investigation, some studies suggest its association with gut health and metabolism (Hoffman and Margolis, 2020). The involvement of the *ADA* enzyme in regulating adenosine levels and the important role of adenosine in immune regulation and inflammatory responses mean that *Turicibacter sanguinis* may indirectly affect *ADA* gene expression or enzyme activity by influencing immune cells or inflammatory signaling pathways. This could result in the negative correlation between *ADA* gene expression and *Turicibacter sanguinis* (*R* = −0.79, *p* = 5.2e-07; Figure 6B).

In addition, the 3-hydroxybutyrate dehydrogenase encoded by *BDH1* participates in reactions of the fatty acid metabolism pathway. It is responsible for converting 3-hydroxybutyrate into acetoacetate, which is an important step in fatty acid oxidation metabolism. This process contributes to the generation of energy supply and the maintenance of normal intestinal cell function (Marks et al., 1992; Otsuka et al., 2020). Meanwhile, *Roseburia hominis*, a beneficial bacteria in the gut microbiota, is primarily involved in the degradation of cellulose and other complex carbohydrates. Bacteria belonging to the *Roseburia* genus play important roles in the human intestinal tract, particularly in the production of short-chain fatty acids, such as propionate, butyrate, and valerate (Tamanai-Shacoori et al., 2017). Therefore, there was a positive correlation between the *BDH1* gene and *Roseburia hominis* (*R* = 0.85, *p* = 1.3e-06; Figure 6B). Finally, the *DRD1* gene encodes the dopamine D1 receptor, which plays a crucial role in the nervous system. The dopamine D1 receptor is a key component in the transmission of dopamine neurotransmitter signals and is involved in regulating numerous functions, such as motor control, emotion, cognition, and reward (Dearry et al., 1990). *Clostridioides difficile* is a pathogenic bacteria that can cause intestinal infections and inflammation. It is typically transmitted through the ingestion of spores or bacteria, and produces toxins that result in symptoms such as diarrhea, abdominal pain, and fever (Sandhu and Mcbride, 2018). There was a positive correlation between *Clostridioides difficile* infection and the *DRD1* gene (*R* = 0.92, *p* = 5.1e-07; Figure 6B). A possible reason for this is that *Clostridioides difficile* infection alters the gut microbiota environment, including the composition and functionality of the microbial community. Such environmental changes may impact the activity of the dopamine signaling pathway and expression of the *DRD1* gene.

## Discussion

In this study, we employed a metagenomic and transcriptomic approach to investigate the gut microbiota community in SPF pigs after stable colonization and to reveal its interaction with the host. By comparing the microbial composition in different GIT sites of SPF pigs, we identified differences in the microbial communities and explored their potential impacts on the host and the mechanisms that underlie these impacts. To the best of our knowledge, this is the first comprehensive study using metagenomics and transcriptomics to investigate structural and functional differences in the gut microbiota among different GIT sites (stomach, small intestine, large intestine, and rectum) of SPF pigs, while also associating the microbiota with host DEGs to explore their interactions.

We employed metagenomics approaches to obtain comprehensive genomic information of the gut microbiota in SPF pigs. This enabled us to gain a more comprehensive understanding of the genetic potential and metabolic functions of the microbiota community and potential mechanisms of its interaction with the host. We observed significant differences in microbial composition among GIT sites, which were closely related to their functions. For example, we found distinct abundance patterns of the two predominant phyla, *Firmicutes* and *Bacteroidetes*, along the GIT: *Firmicutes* abundance decreased in the rectum direction along the GIT (Figure 3A), while *Bacteroidetes* exhibited the opposite pattern (Figure 3A). The high gastric acid and bile salt concentrations in the stomach and upper intestine provide conditions favorable for the survival and proliferation of *Firmicutes*, leading to its increased abundance, while the lower oxygen levels and presence of abundant fermentation substrates in the middle and lower intestines create a suitable environment for the growth of *Bacteroidetes*, potentially resulting in its higher abundance. This contrasting pattern may reflect the adaptability and functional specialization of different microbial communities to different intestinal segments, thereby maintaining the stability and functional balance of the entire GIT. We also discovered the presence of abundant cellulose-degrading microbial communities, such as *Prevotella*, in the large intestine and rectum of SPF pigs, highlighting their important role in the degradation of cellulose and other complex carbohydrates, this suggests that the genus *Prevotella* also includes starch-degrading bacteria. In contrast, the small intestine harbored other microbial groups involved in protein and fat degradation, nutrient absorption, and other essential metabolic processes. These results indicate functional differences in microbial composition among different GIT sites.

After annotation, we identified *Porcine mastadenovirus A* virus in the ileum. We propose three hypotheses to explain its presence. Firstly, the unique anatomical and physiological characteristics of the ileum provide an ideal environment for fecal retention and pathogen proliferation. Secondly, *Porcine mastadenovirus A* may be transmitted through the fecal-oral route. Considering the virus’s mode of transmission, we speculate that the ileum is the site where the virus remains for the longest duration, facilitating its replication and spread. Lastly, the ileal microenvironment is conducive to viral growth. Compared with other intestinal sites, the ileum exhibits a higher pH and electrolyte concentrations that are favorable for the replication of *Porcine mastadenovirus A*.

We annotated the bacterial protein-coding genes and compared them with the KEGG, eggNOG, CAZy, and CARD databases to assess the functionality of the GIT sites and their associated microbial ecology. Surprisingly, we found the functional profiles of the gut microbiota to be similar using KEGG and eggNOG. The gut microbiota in the small intestine of SPF pigs were enriched for nucleotide metabolism, xenobiotic biodegradation and metabolism and digestive system, while the microbiota in the large intestine were enriched for amino acid metabolism, secondary metabolite biosynthesis, and energy metabolism (Supplementary Figures S5, S6A,E). Furthermore, we observed similar differences in the functional enrichment of host DEGs (Figures 5C,D; Supplementary Figure S7). This finding indicates a co-evolutionary trend between the gut microbiota and the host. In addition, we observed significant differences in six CAZyme families across different sites of the GIT. Moreover, different antimicrobial resistance genes within the GIT exhibited variations in resistance mechanisms and best match antibodies, indicating their distinct roles in different GIT sites.

By combining metagenomics with transcriptomics, we revealed interactions between the intestinal microbiota and their impacts on host gene expression. By measuring the mRNA levels in host cells, we observed differences in gene expression between the small and large intestines (Figure 5B). Notably, the distribution of gene expression within the small intestine was not uniform; there were significant differences between the ileum and the other two segments (Figure 5B). Functional enrichment analysis revealed immune-related pathways and terms in the small intestinal tissue, as indicated by both KEGG and GO enrichment analyses (Figure 5C; Supplementary Figure S7A). This suggests that the ileum plays a crucial role as an immune barrier. The ileal mucosa contains numerous lymphoid tissues and immune cells, including Peyer’s patches, lymphoid follicles, and intestinal epithelial cells (Mörbe et al., 2021). Peyer’s patches are lymphoid follicles on the ileal mucosa consisting of aggregated lymphoid cells. These follicles contain a large number of lymphocytes, including B cells and T cells, which are key components of the immune response. Upon exposure to pathogens, these lymphocytes can recognize and initiate appropriate immune reactions, including antibody production and activation of cellular immunity (Carrasco Garcia et al., 2020). Furthermore, the ileum contains a substantial population of intestinal epithelial cells that contribute to immune responses. This immune response is essential for maintaining intestinal health and the normal functioning of the overall immune system (Rath and Haller, 2022). Finally, by analyzing the association between microbiota and host genes, we discovered that the presence of microbiota can regulate host immune responses, metabolic pathways, and other crucial biological processes. This indicates that changes in the microbial community can have a significant impact on the host’s physiological status.

we identified some gut bacteria that are closely associated with host gene expression, and these findings have important implications for future applications. For example, we observed a negative correlation between the expression of *Prevotella dentalis* and the gene *TCN1*, which is related to vitamin B12 metabolism. This finding may contribute to the assessment of vitamin B12 absorption and utilization in the gut. The expression of *Turicibacter sanguinis* was negatively correlated with the immune-regulatory gene *ADA*, suggesting that microorganisms may indirectly regulate *ADA* gene expression by influencing immune cells or inflammatory pathways. The expression of *Roseburia hominis* was positively correlated with the gene *BDH1*, providing new clues for studying intestinal energy metabolism. Additionally, we found a positive correlation between *Clostridioides difficile* infection and the expression of the neuro-related gene *DRD1*. This finding may reveal the impact of gut microbiota on the nervous system, providing a novel perspective for understanding the relationship between gut microbiota and neurological disorders.

This study has some limitations. Firstly, we only investigated a limited number of SPF pig samples. Expanding the sample size would contribute to a more comprehensive understanding of the diversity and functionality of the gut microbiota in SPF pigs. Secondly, while we focused on the composition of the microbial community and changes in host gene expression, further research is needed to understand the specific mechanisms of interaction between the microbiota and the host.

## Conclusion

In this study, we investigated the gut microbiota of SPF Bama females and their interactions with the host. We found significant differences in microbial diversity and functions across different sites of the gastrointestinal tract (GIT). The large intestine and rectum exhibited higher microbial abundances and functions related to polysaccharide fermentation. Additionally, we correlated host gene expression with microbial communities, revealing potential functional interactions between the microbiota and the host, which would be an expected outcome of co-evolution. Our findings highlight the influence of the microbiota on gut function, including immune responses, metabolism, and neural signaling. Overall, this study provides valuable insights into the gut microbiota-host interactions in SPF pigs, contributing to our understanding of pig physiology and husbandry.

In conclusion, our study sheds light on the gut microbiota composition and its interaction with the host in SPF pigs. These findings have implications for animal husbandry, life science research, and bioproduct production.

## Data availability statement

The datasets presented in this study can be found in the Genome Sequence Archive (GSA), accession numbers CRA014067 and CRA014068.

## Ethics statement

The animal studies were approved by the Experimental Animal Ethics Committee, Chongqing Academy of Animal Science. The studies were conducted in accordance with the local legislation and institutional requirements. Written informed consent was obtained from the owners for the participation of their animals in this study.

## Author contributions

MW: Formal analysis, Methodology, Software, Visualization, Writing – original draft, Conceptualization, Data curation, Investigation, Project administration, Resources, Validation. SC: Data curation, Formal analysis, Methodology, Software, Visualization, Validation, Writing – original draft. YZ: Formal analysis, Investigation, Validation, Writing – original draft. YL: Investigation, Methodology, Validation, Writing – original draft. CT: Data curation, Formal analysis, Software, Writing – original draft. JZ: Conceptualization, Funding acquisition, Software, Validation, Writing – original draft. JS: Formal analysis, Investigation, Methodology, Resources, Writing – original draft. XiL: Project administration, Software, Supervision, Visualization, Writing – original draft. YD: Investigation, Software, Writing – original draft. LL: Conceptualization, Project administration, Supervision, Writing – original draft. KL: Investigation, Methodology, Validation, Writing – original draft. YN: Investigation, Methodology, Writing – original draft. XuL: Investigation, Supervision, Validation, Writing – review & editing. ML: Supervision, Writing – review & editing, Funding acquisition. LG: Writing – review & editing, Supervision. JM: Investigation, Methodology, Validation, Writing – review & editing.
