# Supplementary material for: Diversity and host interaction of the gut microbiota in specific pathogen-free pigs
Source: Front Microbiol. 2024 May 10;15:1402807. doi: 10.3389/fmicb.2024.1402807 (PMC11122924; doi:10.3389/fmicb.2024.1402807)
Supplement: Supplementary file 1 [file Data_Sheet_1.docx]

**Supplementary materials**

**Supplementary Table 1. Distribution of metagenomic sequencing samples.**

**Supplementary Table 2. Distribution of transcriptome sequencing samples.**

**Supplementary Table 3. The distribution of taxonomic annotations of SPF pig species at different levels.**

**Supplementary Table 4. The relative abundance of top 20 phyla in SPF pig GIT.**

**Supplementary Fig. 1 Percentage composition of each part of the ORFs.**

**Supplementary Fig. 2 Overview of GIT microbiota in SPF pigs.**

**Supplementary Fig. 3 Diversity and distribution of GIT microbiota in SPF pigs.**

**Supplementary Fig. 4 Biomarker analysis of different sites in the GIT of SPF pigs.**

**Supplementary Fig. 5 Significant differences in KEGG functional profiles between the small intestine and large intestine** **microbial genes of SPF pigs.**

**Supplementary Fig. 6 The microbial genes in SPF pigs exhibit different functions along the GIT.**

**Supplementary Fig. 7 Distinct patterns of GO enrichment for DEGs in the small intestine and large intestine of SPF pigs.**

**Supplementary Table 1. Distribution of metagenomic sequencing samples.**

| **Sample ID** | **Raw Reads** | **Clean Reads** | **GC Content(%)** | **Source** | **Group** |
| --- | --- | --- | --- | --- | --- |
| SPF1_Wcm | 80,730,894 | 77,475,960 | 41.39 | Stomach | Stomach |
| SPF2_Wcm | 90,020,580 | 88,622,030 | 43.14 |  |  |
| SPF3_Wcm | 97,530,982 | 94,827,820 | 42.85 |  |  |
| SPF4_Wcm | 133,739,534 | 131,047,112 | 42.6 |  |  |
| SPF5_Wcm | 65,869,424 | 65,410,168 | 41.02 |  |  |
| SPF6_Wcm | 73,536,122 | 72,715,178 | 42.8 |  |  |
| SPF1_Scm | 91,631,940 | 90,308,492 | 43.66 | Duodenum | Small intestine |
| SPF2_Scm | 73,748,490 | 72,950,464 | 42.1 |  |  |
| SPF3_Scm | 80,926,124 | 79,746,734 | 43.28 |  |  |
| SPF4_Scm | 69,938,474 | 69,307,196 | 42.9 |  |  |
| SPF5_Scm | 99,975,672 | 99,049,120 | 43.14 |  |  |
| SPF6_Scm | 91,181,492 | 87,882,204 | 42.98 |  |  |
| SPF1_Kcm | 79,237,136 | 77,287,112 | 42.98 | Jejunum |  |
| SPF2_Kcm | 98,760,686 | 97,394,518 | 42.94 |  |  |
| SPF3_Kcm | 82,960,312 | 80,721,926 | 42.97 |  |  |
| SPF4_Kcm | 102,381,734 | 100,438,542 | 42.53 |  |  |
| SPF5_Kcm | 101,223,328 | 99,574,572 | 43.03 |  |  |
| SPF6_Kcm | 87,500,460 | 86,069,884 | 42.77 |  |  |
| SPF1_Hcm | 83,750,478 | 82,756,302 | 42.87 | Ileum |  |
| SPF2_Hcm | 98,719,268 | 97,646,892 | 39.84 |  |  |
| SPF3_Hcm | 65,503,094 | 64,745,790 | 42.87 |  |  |
| SPF4_Hcm | 65,755,948 | 64,922,240 | 42.75 |  |  |
| SPF5_Hcm | 74,793,448 | 73,976,872 | 42.79 |  |  |
| SPF6_Hcm | 93,716,728 | 92,618,348 | 44.15 |  |  |
| SPF1_Mcm | 88,413,452 | 87,726,074 | 46.91 | Cecum | Large intestine |
| SPF2_Mcm | 91,197,544 | 90,504,136 | 46.94 |  |  |
| SPF3_Mcm | 103,207,424 | 101,799,830 | 45.4 |  |  |
| SPF4_Mcm | 86,164,934 | 84,493,392 | 46.99 |  |  |
| SPF5_Mcm | 109,924,058 | 108,574,636 | 48.07 |  |  |
| SPF6_Mcm | 73,841,024 | 73,370,306 | 48.08 |  |  |
| SPF1_Jcm | 90,900,756 | 90,254,318 | 44.72 | Colon |  |
| SPF2_Jcm | 104,825,628 | 103,917,954 | 45.84 |  |  |
| SPF3_Jcm | 75,561,276 | 74,462,574 | 42.99 |  |  |
| SPF4_Jcm | 85,819,442 | 84,787,460 | 45.25 |  |  |
| SPF5_Jcm | 108,767,700 | 107,912,544 | 46.95 |  |  |
| SPF6_Jcm | 102,747,108 | 101,905,558 | 48.65 |  |  |
| SPF1_Zcm | 96,591,848 | 95,156,652 | 47.14 | Rectum | Rectum |
| SPF2_Zcm | 90,194,348 | 88,938,964 | 47.37 |  |  |
| SPF3_Zcm | 81,872,148 | 80,706,624 | 46.04 |  |  |
| SPF4_Zcm | 74,507,474 | 74,182,230 | 47.11 |  |  |
| SPF5_Zcm | 109,854,828 | 109,360,632 | 48.18 |  |  |
| SPF6_Zcm | 66,192,874 | 65,953,640 | 48.25 |  |  |

**Supplementary Table 2. Distribution of transcriptome sequencing samples.**

| **Sample ID** | **Raw Reads** | **Clean Reads** | **GC Content(%)** | **Source** | **Group** |
| --- | --- | --- | --- | --- | --- |
| SPF1_ST | 71,620,216 | 70,772,058 | 51.76 | Duodenum | Small intestine |
| SPF2_ST | 54,173,222 | 53,758,843 | 51.34 |  |  |
| SPF3_ST | 73,083,007 | 72,598,470 | 51.55 |  |  |
| SPF4_ST | 41,493,148 | 41,095,029 | 51.22 |  |  |
| SPF5_ST | 79,476,407 | 78,949,882 | 51.69 |  |  |
| SPF6_ST | 45,815,063 | 45,132,289 | 51.95 |  |  |
| SPF1_KT | 45,036,251 | 44,594,113 | 51.18 | Jejunum |  |
| SPF2_KT | 64,207,080 | 63,299,351 | 51.97 |  |  |
| SPF3_KT | 57,051,922 | 56,662,645 | 51.66 |  |  |
| SPF5_KT | 61,263,601 | 60,766,592 | 51.7 |  |  |
| SPF6_KT | 54,458,007 | 53,837,741 | 51.75 |  |  |
| SPF1_HT | 71,185,989 | 70,422,695 | 51.5 | Ileum |  |
| SPF2_HT | 63,058,859 | 62,680,358 | 51.41 |  |  |
| SPF3_HT | 69,394,441 | 68,550,938 | 52.41 |  |  |
| SPF4_HT | 66,301,298 | 65,757,567 | 51.28 |  |  |
| SPF5_HT | 52,660,511 | 52,105,925 | 52.14 |  |  |
| SPF6_HT | 45,674,134 | 44,905,940 | 47.35 |  |  |
| SPF1_MT | 59,957,246 | 59,192,806 | 51.62 | Cecum | Large intestine |
| SPF2_MT | 63,096,951 | 62,454,238 | 51.47 |  |  |
| SPF4_MT | 41,605,047 | 41,305,095 | 50.75 |  |  |
| SPF5_MT | 64,045,509 | 63,411,114 | 52.08 |  |  |
| SPF6_MT | 70,510,059 | 69,127,678 | 51.72 |  |  |
| SPF1_JT | 63,269,504 | 62,689,289 | 50.75 | Colon |  |
| SPF2_JT | 67,398,698 | 66,659,125 | 51.15 |  |  |
| SPF3_JT | 81,396,918 | 80,775,018 | 51.56 |  |  |
| SPF4_JT | 59,747,100 | 59,293,993 | 50.78 |  |  |
| SPF5_JT | 73,370,111 | 72,338,840 | 52.64 |  |  |
| SPF6_JT | 46,948,365 | 46,126,776 | 51.66 |  |  |

**Supplementary Table 3. The distribution of taxonomic annotations of SPF pig species at different levels.**

| **Taxonomy** | **Number** |
| --- | --- |
| Kingdom | 4 |
| Phylum | 61 |
| Class | 113 |
| Order | 226 |
| Family | 470 |
| Genus | 1646 |
| Species | 5421 |

**Supplementary Table 4. The relative abundance of top 20 phyla in SPF pig GIT.**

| **Phylum** | **Stomach** | **Small intestine** | **Large intestine** | **Rectum** |
| --- | --- | --- | --- | --- |
| Firmicutes | 0.733124 | 0.729387 | 0.618539 | 0.436429 |
| Proteobacteria | 0.215909 | 0.133794 | 0.094455 | 0.110775 |
| Bacteroidetes | 0.025506 | 0.046484 | 0.172403 | 0.320302 |
| Unassigned | 0.016375 | 0.034046 | 0.078446 | 0.091368 |
| Preplasmiviricota | 0.00026 | 0.033095 | 0.000335 | 0.000103 |
| Actinobacteria | 0.004102 | 0.01496 | 0.024652 | 0.027534 |
| Ascomycota | 0.002497 | 0.002927 | 0.001097 | 0.001745 |
| Tenericutes | 0.000357 | 0.001207 | 0.000789 | 0.00086 |
| Cyanobacteria | 0.00049 | 0.000461 | 0.001204 | 0.001307 |
| Spirochaetes | 9.41E-05 | 0.000516 | 0.001247 | 0.001548 |
| Fusobacteria | 0.000145 | 0.000642 | 0.000874 | 0.000965 |
| Planctomycetes | 0.000107 | 0.000147 | 0.001081 | 0.00132 |
| Euryarchaeota | 8.21E-05 | 0.000287 | 0.000864 | 0.001024 |
| Apicomplexa | 8.15E-05 | 0.000776 | 0.000448 | 0.000564 |
| Uroviricota | 0.000541 | 0.000278 | 0.00026 | 0.000191 |
| Deinococcus-Thermus | 3.79E-05 | 4.27E-05 | 0.000467 | 0.000619 |
| Verrucomicrobia | 3.67E-05 | 0.000306 | 0.000412 | 0.000507 |
| Chloroflexi | 2.85E-05 | 3.82E-05 | 0.000432 | 0.000516 |
| Thermotogae | 3.42E-05 | 8.84E-05 | 0.000286 | 0.000428 |
| Synergistetes | 1.60E-05 | 1.31E-05 | 0.000262 | 0.000253 |
| Other | 0.000176 | 0.000504 | 0.001446 | 0.001639 |


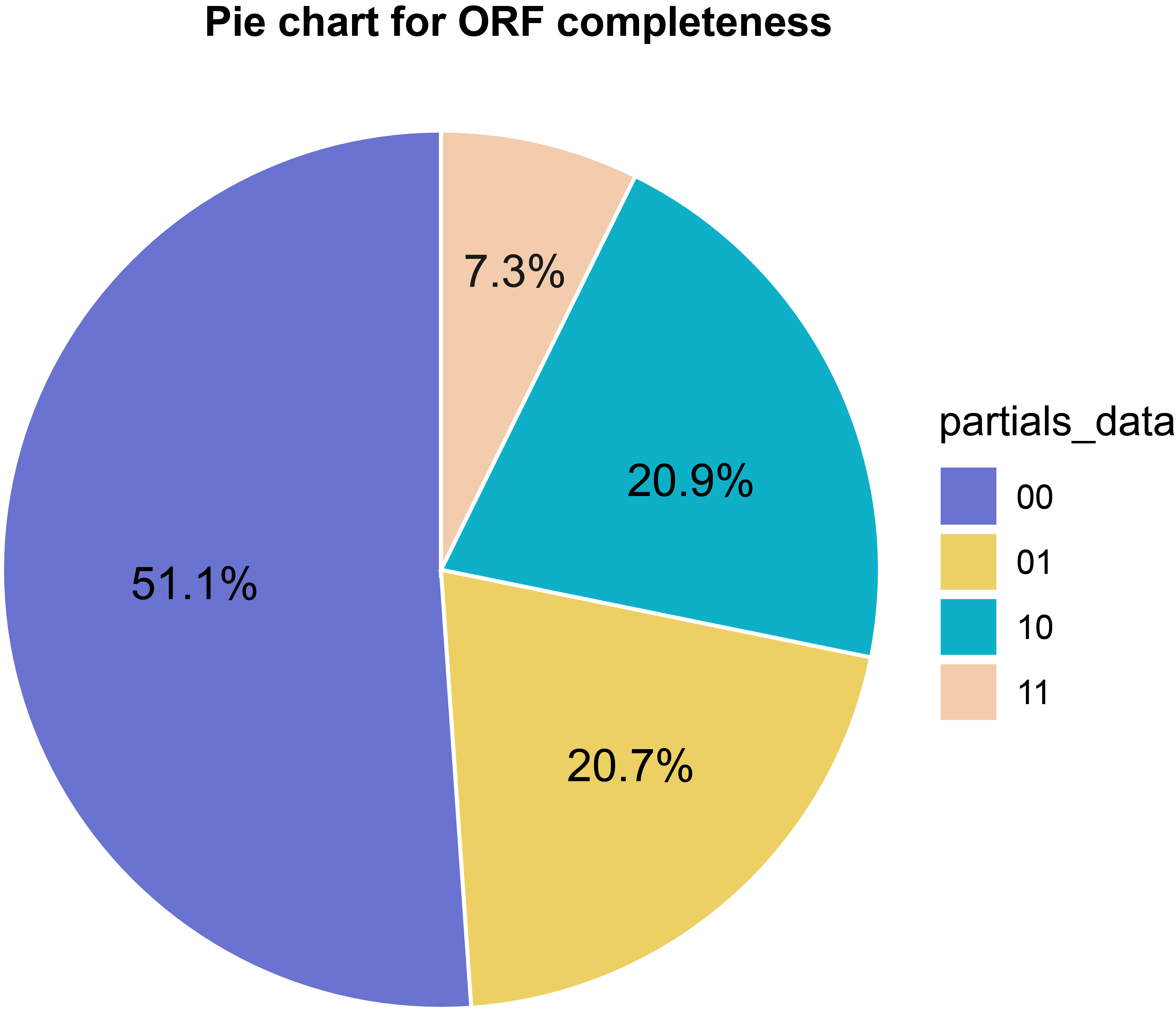


**Supplementary Fig. 1 Percentage composition of each part of the ORFs.** ORFs were predicted using metaProdigal, resulting in a total of 1,213,609 ORFs. In the pie chart, partial=00 represents complete ORFs (620,325 in total, accounting for 51.1%), 01 represents ORFs lacking stop codons (251,055 in total, accounting for 20.7%), 10 represents ORFs lacking start codons (253,636 in total, accounting for 20.9%), and 11 represents ORFs with missing start and stop codons on both ends (88,593 in total, accounting for 7.3%).


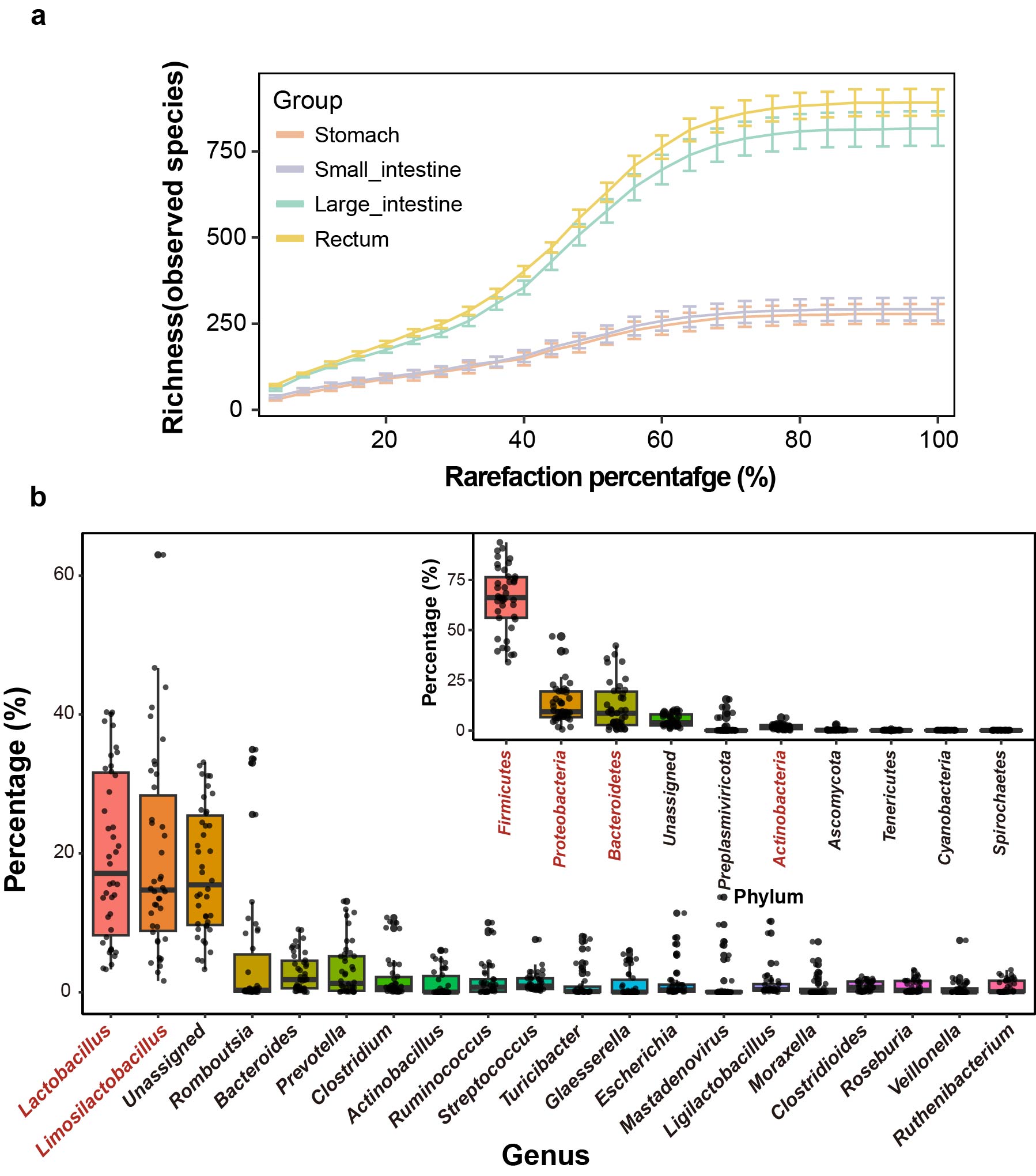


**Supplementary Fig. 2 Overview of GIT microbiota in SPF pigs. a** Rarefaction curve of species in GIT microbiota of SPF pigs. As sequencing depth increases, the rarefaction curve of detected species from the GIT microbiota reaches a saturation stage. Each error bar represents the standard error. The number of replicate samples in the figure is as follows: Stomach (orange, n= 6), Small intestine (purple, n= 18), Large intestine (green, n = 12), and Rectum (yellow, n = 6). **b** Distribution of GIT microbiota in SPF pigs at different taxonomic levels. The box plot presents the top 20 genera and 10 phyla with the highest abundance (top right corner). Y-axis: the percentage of species abundance in relation to the total microbial abundance in the sample. The box plot displays the median, 25th and 75th percentiles, solid lines represent the minimum and maximum values, and points outside the whiskers represent outliers. The phyla and genera of special interest are marked in red.

­­­­



**Supplementary Fig. 3 Diversity and distribution of GIT microbiota in SPF pigs.** Alpha diversity of GIT microbiota in SPF pigs along **a** Group and **b** Source based on the Shannon index. Different colored boxes represent different sites of the GIT: stomach (orange, n = 6), small intestine (purple, n = 18), large intestine (green, n = 12), and rectum (yellow, n = 6). Pairwise Wilcoxon rank-sum test was used to compare the groups. Box plots display the median, 25th, and 75th percentiles, solid lines represent the minimum and maximum values, and points outside the whiskers of the box plot indicate outliers. Principal Coordinate Analysis (PCoA) based on the Bray-Curtis distance of samples from **c** four Groups and **d** seven Sources revealed the beta diversity of GIT microbiota in SPF pigs. **e** Composition of species-level microbiota in SPF pigs along different Sources of the gastrointestinal tract. X-axis: different sources (stomach, duodenum, jejunum, ileum, cecum, colon, and rectum), Y-axis: the relative abundance of microbiota. Significance levels: ns *P* ≥ 0.05, **P* < 0.05, ***P* < 0.01, ****P* < 0.001, *****P* < 0.0001.


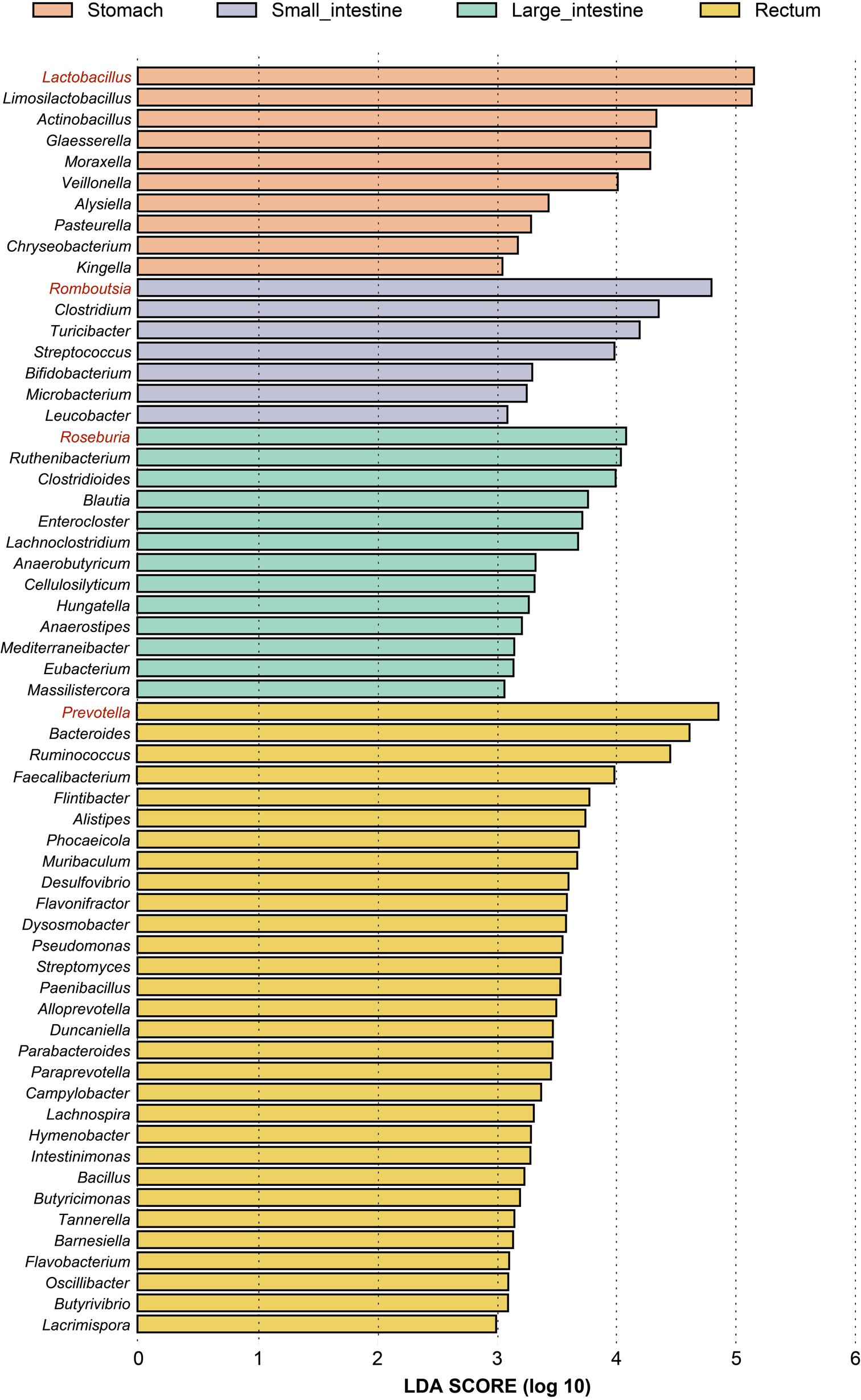


**Supplementary Fig. 4 Biomarker analysis of different sites in the GIT of SPF pigs.** Using linear discriminant analysis effect size (LEfSe) and linear discriminant analysis (LDA) with a threshold of LDA score > 3, 52 taxa with significant abundance differences were identified at the genus level. Different colors in the figure represent different Groups: stomach (orange, n = 6), small intestine (purple, n = 18), large intestine (green, n = 12), and rectum (yellow, n = 6). Biomarkers of particular interest for each group are marked in red.


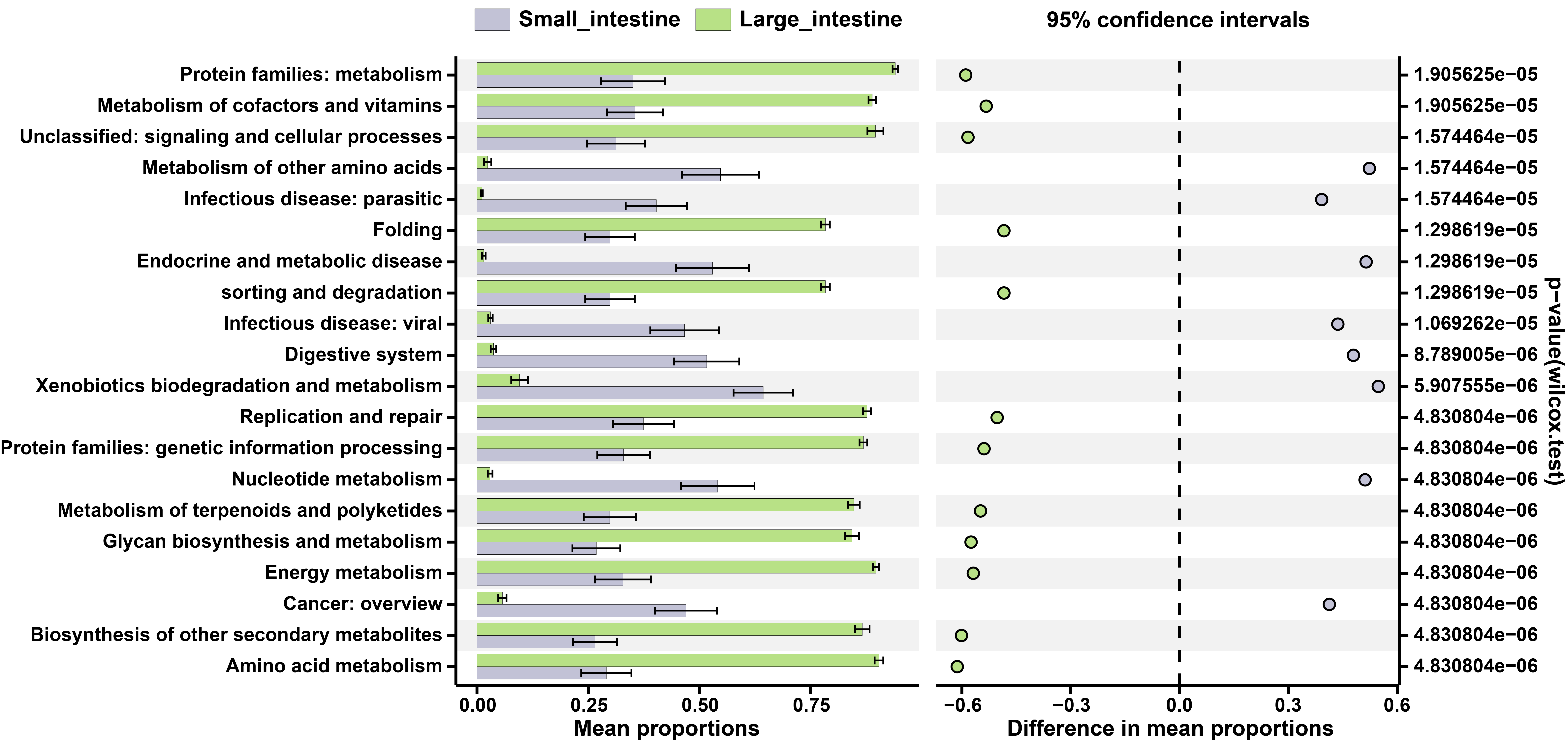


**Supplementary Fig. 5 Significant differences in KEGG functional profiles between the small intestine and large intestine microbial genes of SPF pigs.** The top 20 differentially abundant KEGG pathways based on functional categories in the small intestine (purple, n=18) and large intestine (green, n=12) microbiota were identified using the Wilcoxon rank-sum test and Storey's multiple testing correction method. The left side of the figure presents a bar plot illustrating the numerical differences between the small intestine and large intestine. The right side of the figure displays a scatter plot showing the percentages of functional pathways in both groups relative to all pathways in the respective group.


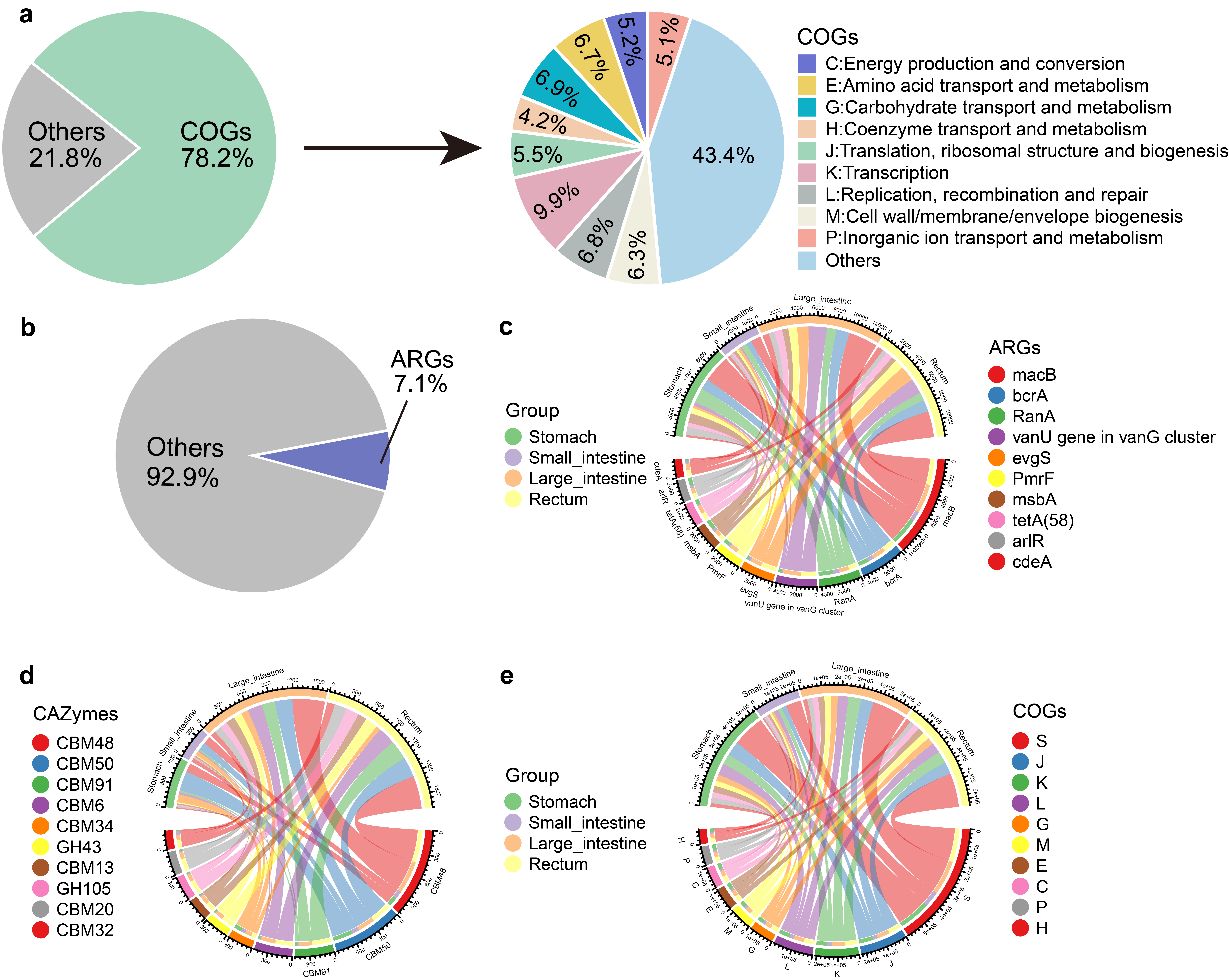


**Supplementary Fig. 6 The microbial genes in SPF pigs exhibit different functions along the GIT.** Functional annotation of microbial protein sequences in SPF pigs. **a** COG annotation results obtained using eggNOG-mapper; the pie chart illustrates the proportion of annotated proteins (left) and the overall categories (right). **b** Protein annotation to the CARD database using DIAMOND; the pie chart displays the proportion of proteins annotatable to ARGs. The Circlize plots demonstrate the abundance distribution patterns of the top 10 annotated **c** ARGs, **d** CAZymes, and **e** COGs across different Groups. S:Function unknown; J:Translation, ribosomal structure, and biogenesis; K: Transcription; L: Replication, recombination and repair; G:Carbohydrate transport and metabolism; M: Cell wall/membrane/envelope biogenesis; N:Cell motility; E:Amino acid transport and metabolism; C:Energy production and conversion; P:Inorganic ion transport and metabolism; H: Coenzyme transport and metabolism。


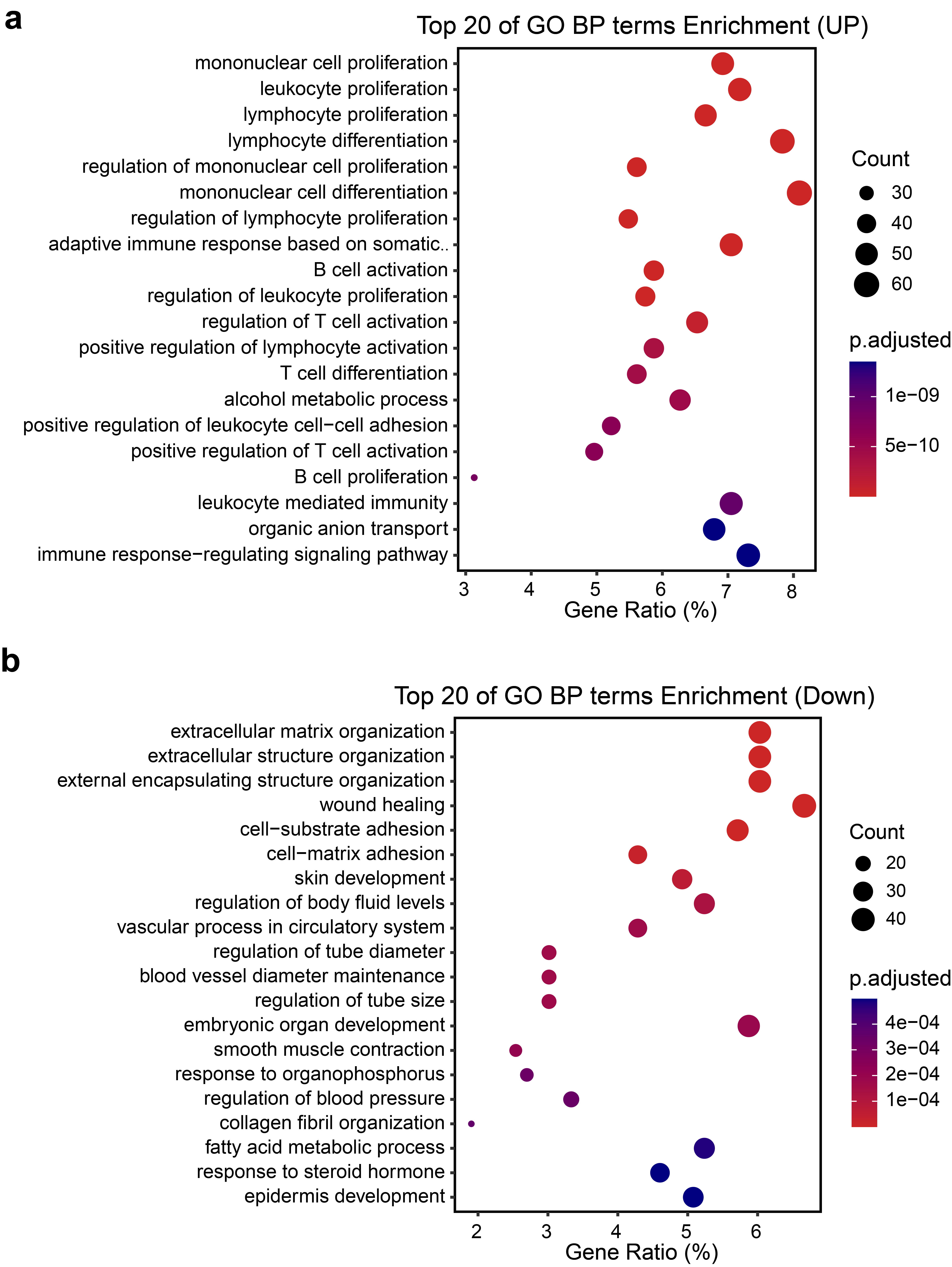
­

**Supplementary Fig. 7 Distinct patterns of GO enrichment for DEGs in the small intestine and large intestine of SPF pigs. a** Bubble plot presents the top 20 enriched gene ontology biological processes (GO BP) for up-expressed genes in the small intestine (adjusted *p* < 0.05). **b** Bubble plot displays the top 20 enriched gene ontology biological processes (GO BP) for down-expressed genes in the small intestine (adjusted *p* < 0.05). X-axis: Gene Ratio (number of genes enriched in a particular term / total number of genes enriched in all terms). The size of the bubbles corresponds to the count of genes.
